# Metabolomic Insights Into the Synergistic Effect of Biapenem in Combination With Xuebijing Injection Against Sepsis

**DOI:** 10.3389/fphar.2020.00502

**Published:** 2020-04-22

**Authors:** Li-Wei Liu, Ying-Ying Shi, Zhuo-Lun Li, Li-Hua Zuo, Meng Tang, Zi-Wei Jing, Hong-Yu Zhao, Peng Xue, Lin Zhou, Qiu-Zheng Du, Xiao-Jian Zhang, Zhi Sun

**Affiliations:** ^1^Department of Pharmacy, the First Affiliated Hospital of Zhengzhou University, Zhengzhou, China; ^2^Henan Key Laboratory of Precision Clinical Pharmacy, Zhengzhou University, Zhengzhou, China; ^3^Henan Engineering Research Center of Clinical Mass Spectrometry for Precision Medicine, Zhengzhou University, Zhengzhou, China; ^4^The First Department of Orthopaedics, Zhengzhou Central Hospital Affiliated to Zhengzhou University, Zhengzhou, China; ^5^Department of Stomatology, the First Afﬁliated Hospital of Zhengzhou University, Zhengzhou, China; ^6^Health Management Centre, the First Afﬁliated Hospital of Zhengzhou University, Zhengzhou, China

**Keywords:** metabolomics, drug combination, synergistic effect, xuebijing injection, biapenem

## Abstract

The drug combination of biapenem (BIPM) and xuebijing injection (XBJ) is commonly applied for the treatment of sepsis in China. However, the potential synergistic mechanism is still enigmatic. There have been no studies focused on the plasma metabolome alterations in sepsis after the intervention of this combination. In this work, an untargeted metabolomics approach was performed by liquid chromatography-mass spectrometry coupled with multivariate statistical analysis to provide new insights into the synergistic effect of BIPM in combination with XBJ. We characterized the metabolic phenotype of sepsis and described metabolic footprint changes in septic rats responding to XBJ and BIPM individually and in combination, in addition to histopathological and survival evaluation. A total of 91 potential biomarkers of sepsis were identified and 32 disturbed metabolic pathways were constructed. Among these biomarkers, 36 metabolites were reversely regulated by XBJ, mainly including glycerophospholipids, sphingolipids, free fatty acids (FFAs), bile acids and acylcarnitines; 42 metabolites were regulated by BIPM, mainly including amino acids, glycerophospholipids, and acylcarnitines; 72 metabolites were regulated after XBJ-BIPM combination treatment, including most of the 91 potential biomarkers. The results showed that the interaction between XBJ and BIPM indeed exhibited a synergistic effect by affecting some key endogenous metabolites, 15 metabolites of which could not be regulated when XBJ or BIPM was used alone. Compared with Model group, 13, 22, and 27 metabolic pathways were regulated by XBJ, BIPM, and XBJ-BIPM combination, respectively. It suggested that many more endogenous metabolites and metabolic pathways were significantly regulated after combination treatment compared with XBJ or BIPM monotherapy. Metabolisms of lipids, amino acids, acylcarnitines, and bile acids were common pathways involved in the synergistic action of XBJ and BIPM. This study was the first to employ metabolomics to elucidate the synergistic effect and decipher the underlying mechanisms of BIPM in combination with XBJ against sepsis. The results provide some support for clinical application of antibiotics in combination with traditional Chinese medicines and have important implications for the treatment of sepsis in clinic.

## Introduction

Sepsis is considered as life-threatening organ dysfunction resulting from dysregulated host response to infection (Singer et al., 2016). Timely administration of antibiotics is the leading line of defense against infection and prescribed as one of the most common medications to treat sepsis. Biapenem (BIPM), a carbapenem antibiotic, exhibits broad spectrum antibacterial activity and has a good curative effect for severe infection (Ikawa et al., 2008). It is widely applied for the treatment of sepsis. However, simply acting on pathogenic microorganisms does not significantly improve survival in patients with sepsis. Antibiotic-mediated endotoxin release and overwhelming inflammatory mediator release play key roles in triggering systemic inflammation reaction syndrome (SIRS) and subsequent multiple organ dysfunction syndrome (MODS) (Lepper et al., 2002). Therefore, as a powerful complement of antibiotic medications, traditional Chinese medicines have been incorporated into routine therapy for the treatment of sepsis (Chinese Society of Critical Care Medicine, 2015).

Xuebijing injection (XBJ), an emerging antiseptic herbal medicine, is prepared from a combination of *Carthamus tinctorius* L., *Paeonia lactiflora* Pall., *Conioselinum anthriscoides* “Chuanxiong”, *Salvia miltiorrhiza* Bunge and *Angelica sinensis* (Oliv.) Diels (**Supplementary Table S1**). It is the only Chinese herb preparation approved by China Food and Drug Administration (CFDA) indicated for sepsis and MODS caused by infections. It has been clinically prescribed for septic patients with good effectiveness on toxic heat flourishing and blood stasis syndrome, as it is considered to possess the efficacies of activating circulation, removing blood stasis, and eliminating toxins (Yin and Li, 2014). In China, approximately 0.8 million patients received treatment with XBJ yearly, about 80% of which were sepsis or septic shock cases (Zhang et al., 2018). In fact, XBJ was most commonly used in combination with antibiotics (e.g., BIPM) for sepsis treatment in clinical practice, instead of being used alone. Numerous clinical studies have shown that addition of XBJ can effectively reduce the 28-day mortality, shorten the length of stay in intensive care units, and improve the physical condition and prognosis of patients with sepsis (Chen and Li, 2013; Gao et al., 2015; Song et al., 2016). The combination of antibiotics and XBJ is one of the medication characteristics of China in clinical treatment of sepsis, and has been showing its advantages in the therapeutic efficacy.

Despite high frequency of antibiotic-XBJ combination and promising clinical effects, the exact synergistic mechanism against sepsis is still enigmatic. Metabolomics provides a cutting-edge approach that measures metabolic alterations in responds to disease process or drug treatment. Characterization of metabolic phenotype facilitates a better understanding of the pathogenesis and interventional mechanism. Mass spectrometry plays an essential role in the metabolic profile analysis. Therefore, this work employs a global metabolomic approach to describe metabolic alterations of septic rats responding to XBJ and BIPM individually and in combination by ultra-high performance liquid chromatography coupled with a Q Exactive hybrid quadrupole-orbitrap high resolution mass spectrometry (UHPLC-Q-Orbitrap HRMS). This work will provide a novel insight into the mechanism of synergistic effect of BIPM in combination with XBJ against sepsis.

## Materials and Methods

### Drugs and Regents

Every 0.3g BIPM (Simcere Pharmaceutical Co., Ltd., Nanjing, China, 92-181004) was dissolved in 30 ml of 0.9% saline for injection. All the endogenous metabolite standards were purchased from Sigma-Aldrich Company (St Louis, MO, USA) and J&K Scientific Ltd. (Beijing, China). Ketoprofen (ESI^-^ internal standard) was purchased from Sigma-Aldrich, and 2-chloro-L-phenylalanine (ESI^+^ internal standard) was purchased from J&K Scientific Ltd. The reference standards of Hydroxysafflor yellow A, Oxypaeoniflorin, Benzoylpaeoniflorin, Senkyunolide I, Succinic acid, Gallic acid, Rosmarinic acid, Caffeic acid, Protocatechuic aldehyde, Protocatechuic acid, Rutin, Salvianic acid A, Chlorogenic acid, and Naringenin were purchased from Chengdu Must Bio-technology Co., Ltd. (Sichuan, China), and the compounds' purities were over 98%. HPLC grade methanol and acetonitrile were obtained from Fisher Scientific (Fair Lawn, NJ, USA). HPLC grade formic acid was supplied by Aladdin Industrial Co., Ltd (Shanghai, China). Ultrapure water was prepared by a Milli-Q purification system (Millipore, Shanghai, China).

XBJ was provided by Tianjin Chase Sun Pharmaceutical Co., Ltd. (Tianjin, China, batch number: 1810221). Per milliliter of XBJ was prepared from the combination of each 0.1 g of *Carthamus tinctorius* L., *Paeonia lactiflora* Pall., *Conioselinum anthriscoides* “Chuanxiong”, *Salvia miltiorrhiza* Bunge and *Angelica sinensis* (Oliv.) Diels, yielding an herb-to-injection ratio of 1:2. The final product of XBJ for intravenous injection is a sterile and nonpyrogenic dosage form. Qualitative and quantitative analysis of XBJ was performed by UHPLC-Q-Orbitrap HRMS. The chromatographic separation was carried out on an ACQUITY UPLC BEH C18 column (50 mm × 2.1 mm, 1.7 μm, Waters, USA) maintained at 40°C. The mobile phase consisted of water containing 0.1% (v/v) formic acid (A) and acetonitrile (B). The gradient elution was set as follows at a flow rate of 0.25 ml/min: 5%–7.5% B at 0–6 min, 7.5%–40% B at 6–30 min, 40%–100% B at 30–50 min, 100% B at 50–55 min, 100%–5% B at 55–56 min, 5% B at 56–60 min. The total ion chromatograms of XBJ were shown in **Supplementary Figure S1**, and a total of 127 components were characterized (**Supplementary Table S2**). We also determined the concentrations of 14 dominating compounds contained in XBJ, the chemical structures of which were shown in **Supplementary Figure S2**. Satisfactory linearity and correlation coefficient were achieved with wide linear range (**Supplementary Table S3**). The precision, repeatability, stability and recovery met requirements (**Supplementary Table S4**).

### Animal Model of Cecal Ligation and Puncture (CLP)-Induced Sepsis

Cecal ligation and puncture (CLP), the most widely used model for experimental sepsis, is considered as the gold standard in sepsis research (Buras et al., 2005; Hubbard et al., 2005). In this work, the rat model of CLP-induced sepsis was performed following previous established protocols (Rittirsch et al., 2009). In brief, rats were anesthetized with ketamine (80 mg/kg) and xylazine (15 mg/kg) by intraperitoneal injection. A midline laparotomy with the incision less than 3 cm was performed to expose the cecum. The cecum was then tightly ligated below the ileocecal valve, and the distal caecum was punctured with a sterile 18G needle. Then, the cecum was gently squeezed to extrude a small amount of intraluminal content. After that, the cecum was returned to abdominal cavity and the incision was sutured. The rats were resuscitated by injecting prewarmed normal saline (37°C, 10 ml/Kg) subcutaneously after the surgical operation. The sham-operated rats underwent the same procedures but the cecum was not subjected to ligation and puncture.

### Animals and Drug Administration

Fifty male Sprague-Dawley (SD) rats (weighing 250–300 g) were purchased from Henan experimental animal center (Zhengzhou, China). These animals were fed with normal diet and water under the controlled temperature (18–22°C) and humidity (55%–65%) to accommodate to the environment for seven days before the experiment. All animal studies were conducted in compliance with the National Institutes of Health Guide for the Care and Use of Laboratory Animals, and the experimental procedures were approved by the Animal Ethics Committee of the first affiliated hospital of Zhengzhou University.

All the rats were randomly divided into five groups. Sham group (n=10): Rats underwent sham surgery in which the cecum was not subjected to ligation and puncture, and intravenous injections of saline *via* tail vein were performed every 12 hours after the sham operation. Model group (n=10): The septic rat model was induced by CLP operation as described above, and saline solution was administrated *via* intravenous injection every 12 hours after the surgery. XBJ group (n=10): Rats received tail vein injection of XBJ (5.25 ml/Kg) every 12 h after CLP operation. BIPM group (n=10): Rats received tail vein injection of BIPM (31.5 mg/Kg) every 12 h after CLP operation. COM group (combined administration of XBJ and BIPM, n=10): Rats received tail vein injection of XBJ (5.25 ml/Kg) right after injection of BIPM (31.5 mg/Kg) every 12 h after CLP operation. The term of the experiment was three continuous days.

### Sample Collection and Preparation

The rats were sacrificed at 72 h after surgical operation. Blood samples were collected from abdominal aorta, and plasma was separated by centrifugation at 3000 rpm for 10 min. The supernatants were stored at -80°C until subsequent analysis. The lung and liver tissues were dissected and fixed in 10% formalin for histopathological examination.

A 100 μl aliquot of plasma was added to a 1.5 ml Eppendorf tube followed by precipitation with 300 μl of methanol (containing 500 ng/ml ketoprofen and 50 ng/ml 2-chloro-L-phenylalanine as internal standard), and the mixture was vortexed for 1 min. The mixture was subsequently centrifuged at 13,000 rpm for 10 min at 4°C, and 200 μl of the supernatant was transferred to an autosampler vial for further UHPLC-Q-Orbitrap HRMS analysis. To ensure data quality of metabolomic profiling, pooled quality control (QC) samples were prepared by mixing 10 μl of plasma from each sample. The pretreatment of QC samples was conducted according to the procedure mentioned above, and one QC sample was injected after every eight samples throughout the run.

### UHPLC-Q-Orbitrap HRMS Analysis

A 5 μl aliquot of the prepared sample was injected into ACQUITY UPLC BEH C18 column (100 mm × 2.1 mm, 1.7 μm, Waters, USA) maintained at 40°C using a Thermo Scientific Dionex UltiMate 3000 UHPLC system for chromatographic separation. The mobile phase consisted of water containing 0.1% (v/v) formic acid (A) and acetonitrile (B). The gradient elution was set as follows at a flow rate of 0.35 ml/min: 5% B at 0–1min, 5%–100% B at 1–9 min, 100% B at 9–12 min, 100%–5% B at 12–12.1 min, 5% B at 12.1–15 min.

The mass spectrometry was performed on Q-Exactive orbitrap system (Thermo Fisher Scientific, San Jose, USA) equipped with a heated electrospray ionization source operated in both positive and negative ion modes. The MS parameters were optimized and set as follows: collision energy at 20, 40, and 60 eV, ion source temperature at 350°C, capillary temperature at 320°C, spray voltage at 3.50 kV for the positive ion mode and 2.8 kV for the negative ion mode, sheath gas flow rate at 40 arb for the positive ion mode and 38 arb for the negative ion mode, auxiliary gas flow rate at 10 arb. Metabolomic profiles were acquired with a mass range of 80–1200 m/z. The full scan spectra and MS/MS data were collected with the resolution of 70,000 and 17,500 FWHM respectively. The samples were injected in random order, and all the mass data were acquired and processed using Thermo Xcalibur 3.0 software.

### Data Processing and Statistical Analysis

The pretreatment of LC-MS raw data was conducted by Thermo Scientific Compound Discoverer 3.0 software. The spectra were selected from input LC-MS data files and retention time alignment was accomplished based on mass tolerance and time shift criteria. Preliminary identification of metabolites was realized by searching databases including ChemSpider, Mass Lists, mzCloud, mzVault, and local database. Multiple nodes such as “Align Retention Times”, “Detect Unknown Compounds”, “Group Unknown Compounds”, “Search Database”, “Fill Gaps”, and “Normalize Areas” were combined to form an untargeted metabolomics workflow for raw data processing.

The data matrix obtained from Compound Discoverer were imported into SIMICA software (version 14.0, Umetrics, Sweden) for multivariate statistical analysis, including unsupervised principal component analysis (PCA), supervised partial least-squares discriminant analysis (PLS-DA) and orthogonal partial least-squares discriminant analysis (OPLS-DA). The PCA analysis was applied to assess the reproducibility and stability of QC samples, and the PLS-DA model was established to describe general separation of samples from five groups. The variables responsible for the discrimination between two groups were identified by OPLS-DA, and permutation test was performed 200 times to assess the risk of overfitting for the OPLS-DA model. In addition to the multivariate statistical method, Student's *t* test was also applied to measure the significance of each metabolite. The metabolites with variable importance in the projection (VIP) values >1.0 and *p* values <0.05 for Model versus Sham were considered as potential biomarkers of sepsis, the structures of which were further confirmed based on available reference standards.

To evaluate the effect of drug intervention, fold changes and *p* values of the potential biomarkers for comparisons of XBJ versus Model, BIPM versus Model, and COM versus Model were calculated respectively. The concentrations of metabolites detected in different groups were expressed as peak areas. Histograms were described to reflect the changes of potential biomarkers across the five groups using GraphPad Prism 8.0.2 software (GraphPad Software Inc., San Diego, USA). Correlation analysis, metabolic pathway analysis, heat map and hierarchical cluster analysis were conducted by MetaboAnalyst (http://www.metaboanalyst.ca/).

## Results

### Histopathological Examination and Survival Analysis

The effects of three different interventions on the histological morphology of lung and liver tissues in septic rats by hematoxylin- eosin (HE) staining were shown in **Supplementary Figure S3**. In the lung tissue of Sham group, the alveolar septum was normal and there was no inflammatory cell infiltration. In the Model group, the alveolar wall was thickened and the blood vessels were congested. In the XBJ and BIPM group, the alveolar walls were less thickened. The histological morphology of lung tissue in COM group was similar to that of Sham group. In the liver tissue sections of Sham group, the hepatic cords were arranged in a radial arrangement. There was no edema in the hepatocytes, and the structure of the hepatic lobule was clear. The Model group showed hepatic cord disorder and cellular swelling of the hepatocyte. Hepatic lobular structure was unclear, and there was a large number of inflammatory cell infiltration. After intervention by XBJ, BIPM, and XBJ-BIPM combination, the hepatic cord structures were acceptable, and the inflammatory cells were reduced. The hepatocytes were mildly edema in XBJ and BIPM groups, and the edema was significantly relieved in the COM group.

Survival analysis showed that the rats in Sham group all survived within 72 h. The survival rates of rats in Model, XBJ, BIPM, and COM groups were 60%, 60%, 70%, and 90%, respectively (**Supplementary Figure S4**). Survival time was prolonged after drug intervention, especially in the COM group. The results indicated that the combination of XBJ and BIPM could improve the survival rate of septic rats.

### Reproducibility and Stability Assessment of the Analytical Strategy

Typical base peak chromatograms (BPCs) of representative samples from five groups were shown in **Supplementary Figure S5**. To characterize the general distribution of all the samples and assess the quality of QC data, unsupervised PCA analysis was performed. The tight cluster of QC samples both in positive and negative (**Supplementary Figures S6A, B**) ion modes demonstrated good reproducibility of analytical method and instrumental stability throughout the run. The reproducibility was also evaluated by the relative standard deviation (RSD) of peak areas across the QC samples, and over 90% of the peaks showed RSD values below 30% (**Supplementary Figures S6C, D**). Moreover, RSD values of ESI^+^ and ESI^-^ internal standards across all the samples were 4.9% and 6.6% respectively. The relative abundance variabilities with respect to injection order for internal standards were presented in **Supplementary Figures S6E, F**. The results provided evidence that our analytical strategy possessed satisfactory reproducibility and stability.

### Metabolic Disturbance Related to Sepsis and Identification of Potential Biomarkers

**Figure 1:**
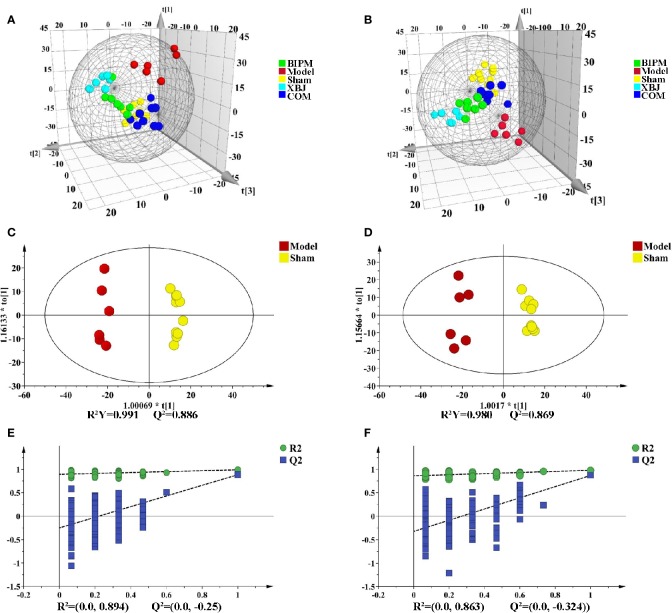
PLS-DA and OPLS-DA score plots in positive and negative ion modes. PLS-DA score plots of different groups in positive **(A)** and negative **(B)** ion modes. OPLS-DA score plots for Model group *vs.* Sham group in positive **(C)** and negative **(D)** ion modes. Permutation tests obtained from Model group *vs.* Sham group in positive **(E)** and negative **(F)** ion modes. PLS-DA, partial least-squares discriminant analysis; OPLS-DA, orthogonal partial least-squares discriminant analysis.

Supervised PLS-DA model was constructed to explore the distribution and tendency of five groups. As shown in **Figures 1A, B**, good separation trend between groups was observed in both positive and negative ion modes. It was obvious that metabolic change occurred in the Model group compared with Sham group. Three different ways of drug intervention exerted different effects by influencing endogenous metabolites, and the COM group was closer to the Sham group than XBJ and BIPM groups. The OPLS-DA model of comparison between Model and Sham group was next performed to characterize the metabolic disturbance related to sepsis. Significant distinctions were obtained with R^2^Y at 0.991 and Q^2^ at 0.886 for ESI^+^ mode (**Figure 1C**), and R^2^Y at 0.980 and Q^2^ at 0.869 for ESI^-^ mode (**Figure 1D**). To assess the validity of the OPLS-DA model, permutation test with 200 measurements was performed and the result (R^2^ = 0.894, Q^2^ = -0.250 for ESI^+^ mode; R^2^ = 0.863, Q^2^ = -0.324 for ESI^-^ mode) indicated that there was no overfitting of the model (**Figures 1E, F**).

With the criteria of VIP values >1.0 and *p* values <0.05, 459 and 426 differential ions were selected for Model *vs.* Sham in ESI^+^ and ESI^-^ mode, respectively. The volcano plot was subsequently performed on the basis of *p* values and fold change values between the two groups. As shown in **Supplementary Figure S7**, the red dots represented metabolites with *p* values <0.05 and fold change values >1.20 (or <0.83). According to accurate m/z and fragmentation information, a total of 91 differential endogenous metabolites were identified, 22 metabolites of which were confirmed by standard references. The MS/MS spectra and fragment ions for some of these metabolites in certain samples compared with standard references were provided in **Supplementary Figure S8**.

As listed in **Table 1**, 63 metabolites elevated and 28 metabolites decreased in Model group. Compared with Sham group, the lipids metabolic disturbance was observed in sepsis including declined lysophosphatidylcholines (LysoPCs) and lysophosphatidylethanolamines (LysoPEs), increased phosphatidylcholines (PCs), elevated free fatty acids (FFAs), and decreased sphingolipids. Altered lipid levels reflected systemic changes resulted from inflammatory response and oxidative stress in sepsis (Sevastou et al., 2013). Significant decreases in LysoPCs and LysoPEs levels in septic rats were consistent with a previous study (Xu et al., 2018). The elevated amino acids, especially the aromatic and sulfur containing amino acids (phenylalanine, tyrosine, and methionine), suggested the imbalance between the hepatic protein synthesis and muscle protein breakdown in energy metabolism of sepsis (Freund et al., 1979). The plasma amino acid pattern was considered as an indication of the severity of metabolic disturbance in sepsis and might be predictive of mortality (Freund et al., 1979). Significantly increased levels of acyl carnitines (hexanoylcarnitine, stearoylcarnitine, oleoylcarnitine, etc.) in Model group indicated enhanced fatty acid β-oxidation in sepsis. In addition, the increased level of N-Acetylneuraminic (Neu5Ac) acid was observed as one of the most significantly changed metabolic markers for sepsis. Neu5Ac was produced by the catalysis of neuraminidase, which played a central role in NF-κB activation and pro-inflammatory responses (Abdulkhalek and Szewczuk, 2013). Multiple organ dysfunction has been implicated in the pathogenesis of sepsis, as indicated by elevated level of creatinine, the biomarker for kidney damage. The increased plasma bile acids might be a consequence of liver injury and dysfunction in the course of sepsis. These disregulated endogenous metabolites were considered as potential biomarkers of sepsis.

**Table 1:** Metabolomic-based potential biomarkers of sepsis and the levels change of metabolites responding to three different interventions.

No.	Metabolites	Theoretical mass (*m/z*)	Measured mass (*m/z*)	Delta (ppm)	RT (min)	Ion Mode	VIP	Fold Change (Model/Sham)	Fold Change (XBJ/Model)	Fold Change (BIPM/Model)	Fold Change (COM/Model)	Classification
1	Lactic acid^#^	89.02441	89.02436	-0.644	1.15	n	1.63	1.83↑^**^	0.55↓^**^	0.55↓^**^	0.59↓^**^	Organic acids
2	Choline	104.10699	104.10700	0.090	0.88	p	1.42	2.15↑^*^	–	0.53↓^*^	0.51↓^*^	Organonitrogen compounds
3	Glyceric acid	105.01933	105.01932	-0.114	1.14	n	1.12	1.57↑^*^	–	–	–	Organic acids
4	Cytosine	112.05053	112.05052	-0.164	0.88	p	1.47	1.81↑^**^	–	0.71↓^*^	0.74↓^*^	Pyrimidines
5	Uracil	113.03455	113.03449	-0.566	1.14	p	1.37	3.41↑^*^	–	–	–	Pyrimidines
6	Creatinine^#^	114.06618	114.06629	0.890	0.86	p	1.36	1.48↑^**^	–	–	0.65↓^**^	other
7	2-Oxovaleric acid	115.04006	115.03997	-0.847	2.48	n	1.24	1.34↑^**^	–	–	–	Organic acids
8	Betaine^#^	118.08625	118.08626	0.041	0.89	p	1.18	0.44↓^**^	–	–	–	other
9	Nicotinamide^#^	123.05528	123.05530	0.086	1.14	p	1.14	0.54↓^*^	–	–	1.78↑^*^	other
10	Pyroglutamic acid^†^	130.04986	130.05000	1.002	0.89	p	1.60	1.47↑^**^	0.72↓^**^	0.71↓^**^	0.75↓^*^	Amino acids
11	4-Oxoproline^†^	128.03531	128.03531	-0.050	1.15	n	1.56	1.74↑^**^	0.67↓^*^	0.59↓^**^	0.63↓^**^	Amino acids
12	Pipecolic acid	130.08625	130.08636	0.806	0.88	p	1.35	1.55↑^**^	–	–	–	Amino acids
13	Creatine^#^	132.07675	132.07684	0.658	0.87	p	1.72	2.45↑^**^	–	0.64↓^*^	0.49↓^**^	Organic acids
14	2-hydroxycaproic acid	131.07136	131.07121	-1.202	3.98	n	1.16	1.85↑^*^	–	0.57↓^*^	0.58↓^*^	Organic acids
15	Threonic acid	135.02989	135.02983	-0.493	0.90	n	1.71	1.77↑^**^	–	0.58↓^**^	0.60↓^**^	Organic acids
16	3-Dehydroxycarnitine	146.11755	146.11763	0.511	0.90	p	1.52	1.58↑^**^	–	–	0.69↓^**^	other
17	Glutamine^#†^	147.07641	147.07649	0.485	0.89	p	1.58	1.49↑^**^	0.71↓^**^	0.72↓^**^	0.70↓^**^	Amino acids
18	β-Ureidoisobutyric acid^†^	147.07641	147.07652	0.689	0.89	p	1.50	1.46↑^**^	0.73↓^*^	0.72↓^**^	0.72↓^**^	Organic acids
19	Lysine^#^	147.11280	147.11284	0.243	0.75	p	1.40	1.58↑^**^	–	–	0.68↓^**^	Amino acids
20	Glutamate^#†^	146.04588	146.04597	0.609	0.90	n	1.33	1.81↑^*^	0.57↓^*^	0.55↓^*^	0.64↓^*^	Amino acids
21	Methionine^#†^	150.05832	150.05843	0.694	1.14	p	1.70	1.64↑^**^	–	0.73↓^**^	0.69↓^**^	Amino acids
22	Allantoin^†^	157.03671	157.03656	-0.977	0.90	n	1.12	1.58↑^*^	0.58↓^*^	–	0.62↓^*^	other
23	3-Dehydrocarnitine	158.08226	158.08223	-0.231	3.89	n	1.07	0.30↓^*^	–	–	–	other
24	2-Hydroxycinnamic acid^#^	165.05462	165.05475	0.784	1.16	p	1.04	1.36↑^*^	–	0.71↓^*^	0.76↓^*^	Organic acids
25	Phenylalanine^#†^	166.08625	166.08633	0.451	1.95	p	1.07	1.34↑^*^	–	0.66↓^*^	0.70↓^*^	Amino acids
26	Uric acid*^#^*^†^	169.03561	169.03566	0.257	1.14	p	1.42	6.08↑^*^	0.30↓^*^	–	0.26↓^*^	Organic acids
27	1-Methylhistidine	170.09240	170.09254	0.805	0.77	p	1.55	2.54↑^*^	–	0.59↓^*^	0.44↓^**^	Amino acids
28	Hippuric acid^#†^	178.05096	178.05099	0.132	3.73	n	1.32	0.19↓^**^	–	–	2.45↑^*^	Organic acids
29	Inositol	179.05611	179.05617	0.328	0.94	n	1.15	0.63↓^*^	–	–	1.56↑^*^	other
30	Tyrosine^#†^	182.08116	182.08131	0.770	1.15	p	1.04	1.36↑^*^	–	0.71↓^*^	0.76↓^*^	Amino acids
31	Phosphorylcholine	184.07332	184.07318	-0.766	7.96	p	1.45	0.71↓^**^	–	1.31↑^*^	1.41↑^**^	Organonitrogen compounds
32	5-Hydroxyisourate	183.01597	183.01587	-0.590	1.14	n	1.53	4.99↑^*^	0.42↓^*^	0.39↓^*^	0.36↓^**^	Organic acids
33	Indoleacrylic acid	186.05605	186.05608	0.151	5.18	n	1.19	0.15↓^**^	2.62↑^*^	–	4.56↑^*^	Indole derivatives
34	N6,N6,N6-Trimethyl-L-lysine	189.15975	189.15982	0.347	0.80	p	1.58	2.29↑^**^	–	0.45↓^**^	0.52↓^**^	Amino acids
35	Citric acid^#^	191.01972	191.01987	0.755	1.12	n	1.15	0.47↓^*^	–	1.81↑^*^	1.90↑^*^	Organic acids
36	Acetyl-L-carnitine^#^	204.12303	204.12308	0.223	1.13	p	1.35	1.45↑^**^	–	–	0.60↓^**^	Acyl carnitine
37	Lipoic acid	207.05079	207.05067	-0.617	0.79	p	1.71	2.39↑^**^	0.69↓^*^	0.70↓^*^	0.54↓^**^	Organic acids
38	N-Acetyl-L-phenylalanine^†^	206.08226	206.08238	0.550	4.40	n	1.08	0.61↓^*^	–	–	–	Amino acids
39	3-Oxododecanoic acid	213.14961	213.14978	0.761	6.98	n	1.44	4.16↑^*^	0.38↓^*^	–	0.40↓^*^	Free fatty acids
40	12-Hydroxydodecanoic acid	215.16526	215.16531	0.196	7.49	n	1.06	1.86↑^*^	–	–	–	Free fatty acids
41	Prolylleucine	229.15466	229.15454	-0.563	0.90	p	1.26	2.75↑^*^	–	–	–	Amino acids
42	Pseudouridine^†^	243.06225	243.06239	0.538	0.90	n	1.43	1.86↑^**^	–	0.65↓^*^	0.59↓^*^	other
43	3-hydroxybutyrylcarnitine	248.14924	248.14923	-0.078	1.15	p	1.13	1.53↑^*^	–	–	0.56↓^**^	Acyl carnitine
44	Hexanoylcarnitine	260.18563	260.18576	0.481	4.37	p	1.68	3.01↑^**^	0.73↓^*^	0.54↓^*^	0.40↓^**^	Acyl carnitine
45	Neuraminic acid^†^	266.08813	266.08823	0.339	0.88	n	1.71	3.03↑^**^	–	0.54↓^*^	0.34↓^**^	Organic acids
46	His-asp	269.08914	269.08789	-4.656	0.89	n	1.63	2.37↑^**^	0.55↓^*^	0.45↓^**^	0.44↓^**^	Amino acids
47	Juniperic acid	271.22786	271.22806	0.707	9.67	n	1.48	1.54↑^**^	0.82↓^*^	–	0.74↓^*^	Free fatty acids
48	Methyl aspartylphenylalaninate^†^	295.12884	295.12888	0.108	3.53	p	1.40	1.79↑^*^	–	0.59↓^*^	0.55↓^*^	Amino acids
49	Sphingosine^#^	300.28970	300.28952	-0.619	7.32	p	1.21	0.32↓^**^	2.16↑^**^	–	2.77↑^*^	Sphingolipids
50	Sphinganine	302.30535	302.30524	-0.384	7.08	p	1.30	0.75↓^**^	1.16↑^*^	–	1.24↑^*^	Sphingolipids
51	Arachidonic acid^#^	303.23295	303.23315	0.648	9.78	n	1.21	1.30↑^**^	0.81↓^*^	–	0.83↓^*^	Free fatty acids
52	N-Acetylneuraminic acid	308.09870	308.09891	0.668	0.95	n	1.70	7.06↑^**^	–	0.37↓^*^	0.20↓^**^	Organic acids
53	Docosahexaenoic acid^#^	327.23295	327.23325	0.906	9.62	n	1.50	1.94↑^**^	0.74↓^*^	–	0.64↓^*^	Free fatty acids
54	Trihydroxy octadecenoic acid	329.23334	329.23315	-0.600	5.90	n	1.36	2.41↑^*^	0.44↓^*^	0.41↓^*^	0.56↓^*^	Free fatty acids
55	Docosapentaenoic acid^#^	329.24860	329.24881	0.627	10.02	n	1.08	1.99↑^*^	0.53↓^*^	–	0.58↓^*^	Free fatty acids
56	3-Indole carboxylic acid glucuronide^†^	336.07248	336.07285	1.072	3.61	n	1.05	0.08↓^*^	–	–	5.13↑^*^	other
57	5-Hydroxy-6-methoxyindole glucuronide^†^	338.08813	338.08853	1.154	3.86	n	1.28	0.07↓^**^	–	–	–	other
58	N-Acetyl-4-O-Acetylneuraminate^†^	350.10926	350.10959	0.917	1.15	n	1.52	11.90↑^*^	–	–	0.11↓^*^	Organic acids
59	Tetradecenoylcarnitine	370.29518	370.29489	-0.797	7.05	p	1.23	2.04↑^**^	1.60↑^*^	–	–	Acyl carnitine
60	Ursodeoxycholic acid^†^	391.28538	391.28583	1.142	7.02	n	1.37	3.05↑^**^	0.41↓^*^	–	0.36↓^**^	Bile acids
61	Deoxycholic acid^#†^	391.28538	391.28580	1.066	7.65	n	1.21	3.21↑^**^	0.31↓^*^	–	0.31↓^*^	Bile acids
62	Hexadecenoylcarnitine	398.32648	398.32642	-0.164	7.54	p	1.42	2.15↑^**^	0.72↓^*^	–	0.64↓^*^	Acyl carnitine
63	Palmitoylcarnitine^#^	400.34213	400.34189	-0.613	7.94	p	1.45	2.23↑^**^	0.74↓^*^	–	0.60↓^*^	Acyl carnitine
64	Linoleyl carnitine	424.34213	424.34198	-0.366	7.70	p	1.37	1.87↑^**^	0.76↓^*^	0.68↓^*^	0.66↓^*^	Acyl carnitine
65	Oleoylcarnitine	426.35778	426.35764	-0.341	8.04	p	1.37	2.28↑^**^	–	–	–	Acyl carnitine
66	Stearoylcarnitine	428.37343	428.37335	-0.200	8.40	p	1.48	2.63↑^**^	–	–	–	Acyl carnitine
67	Chenodeoxyglycocholic acid^†^	448.30684	448.30734	1.100	6.83	n	1.44	4.79↑^**^	0.37↓^*^	–	0.31↓^**^	Bile acids
68	LysoPE(18:0)^†^	482.32411	482.32391	-0.427	8.05	p	1.22	0.31↓^**^	–	1.89↑^*^	2.85↑^**^	Glycerophospholipids
69	LysoPC(O-16:0)	482.36050	482.36060	0.205	8.73	p	1.10	0.80↓^*^	–	1.34↑^*^	1.32↑^*^	Glycerophospholipids
70	LysoPC(16:0)	496.33976	496.33972	-0.092	8.49	p	1.21	0.79↓^**^	–	–	1.16↑^*^	Glycerophospholipids
71	LysoPE(20:4)^†^	502.29281	502.29251	-0.609	8.07	p	1.31	1.57↑^**^	–	–	0.69↓^*^	Glycerophospholipids
72	LysoPE(20:3)^†^	504.30846	504.30826	-0.408	8.38	p	1.17	0.21↓^*^	–	–	3.74↑^**^	Glycerophospholipids
73	LysoPC(17:0)	510.35541	510.35541	-0.012	8.93	p	1.48	0.45↓^**^	1.17↑^*^	1.55↑^*^	2.13↑^**^	Glycerophospholipids
74	LysoPC(18:3)	518.32411	518.32416	0.085	7.73	p	1.34	0.75↓^**^	–	–	–	Glycerophospholipids
75	LysoPC(18:2)	520.33976	520.33984	0.143	8.11	p	1.36	0.68↓^**^	–	–	1.38↑^*^	Glycerophospholipids
76	LysoPC(18:1)	522.35541	522.35553	0.218	8.68	p	1.16	0.66↓^*^	1.27↑^*^	1.28↑^*^	1.50↑^*^	Glycerophospholipids
77	LysoPE(22:6)^†^	526.29281	526.29272	-0.182	8.02	p	1.13	0.75↓^*^	1.98↑^**^	1.64↑^*^	1.74↑^**^	Glycerophospholipids
78	LysoPC(20:5)	542.32411	542.32410	-0.029	7.64	p	1.41	0.68↓^**^	–	1.27↑^*^	1.31↑^*^	Glycerophospholipids
79	LysoPC(20:4)	544.33976	544.33936	-0.746	8.09	p	1.68	0.65↓^**^	–	1.35↑^**^	1.43↑^**^	Glycerophospholipids
80	LysoPC(20:2)	548.37106	548.37091	-0.284	8.86	p	1.21	0.42↓^**^	1.34↑^*^	1.63↑^*^	2.25↑^*^	Glycerophospholipids
81	Taurolithocholic acid-3-sulfate	562.25138	562.25158	0.356	13.50	n	1.74	13.29↑^**^	–	–	–	Bile acids
82	LysoPC(22:4)	572.37106	572.37103	-0.063	8.70	p	1.23	0.60↓^**^	1.27↑^*^	1.34↑^*^	1.52↑^**^	Glycerophospholipids
83	LysoPI(20:4)	619.28888	619.28912	0.377	13.50	n	1.66	0.52↓^**^	1.61↑^**^	1.51↑^*^	1.62↑^*^	Glycerophospholipids
84	PC(16:0/18:2)	758.56943	758.56940	-0.041	13.42	p	1.29	1.51↑^**^	–	–	–	Glycerophospholipids
85	PC(16:0/18:1)	760.58508	760.58337	-2.250	13.48	p	1.50	0.38↓^**^	–	–	–	Glycerophospholipids
86	PC(18:3/18:2)	780.55378	780.55139	-3.064	13.42	p	1.39	2.38↑^*^	–	–	0.63↓^*^	Glycerophospholipids
87	PC(18:1/18:2)	784.58508	784.58270	-3.035	13.52	p	1.62	4.31↑^**^	0.54↓^*^	0.61↓^*^	0.61↓^*^	Glycerophospholipids
88	PC(18:0/18:2)	786.60073	786.60071	-0.027	13.44	p	1.25	1.56↑^**^	0.81↓^*^	0.75↓^*^	0.82↓^*^	Glycerophospholipids
89	PC(16:0/22:6)	806.56943	806.56696	-3.064	13.42	p	1.08	1.21↑^*^	–	–	–	Glycerophospholipids
90	PC(16:0/22:5)	808.58508	808.58246	-3.242	13.41	p	1.24	1.91↑^**^	0.69↓^*^	0.71↓^*^	0.67↓^*^	Glycerophospholipids
91	PC(18:0/22:6)	834.60073	834.60034	-0.469	13.53	p	1.39	3.12↑^*^	–	–	–	Glycerophospholipids

^#^metabolites were identified by reference standards; ^†^metabolites were detected in both negative and positive ion modes; ^*^p values < 0.05; ^**^p values < 0.01; RT, retention time; VIP, variable importance in the projection obtained from Model group vs. Sham group; XBJ, xuebijing injection; BIPM, biapenem; COM, combination of xuebijing injection and biapenem; LysoPE, lysophosphatidylethanolamine; LysoPC, lysophosphatidylcholine; LysoPI, lysophosphatidylinositol; PC, phosphatidylcholine; p, positive ion mode; n, negative ion mode.

### Metabolomic Insights Into the Interventional Effects of XBJ, BIPM, and XBJ-BIPM Combination

**Figure 2:**
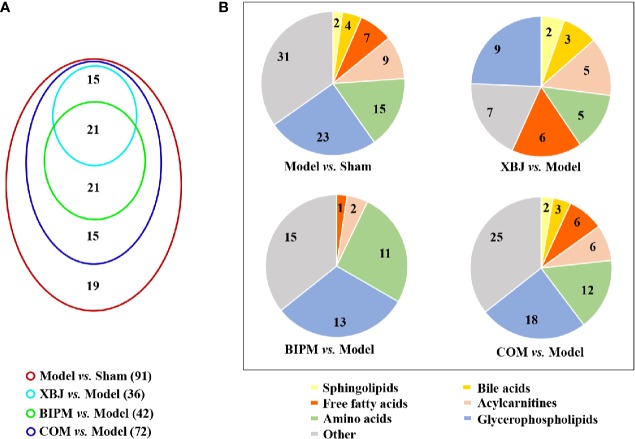
**(A)** Venn diagram showing the number of regulated metabolites for different comparisons. **(B)** Pie charts showing the classification of regulated metabolites for different comparisons. XBJ, xuebijing injection; BIPM, biapenem; COM, combination of xuebijing injection and biapenem.

The OPLS-DA models for XBJ *vs.* Model, BIPM *vs.* Model, and COM *vs.* Model were constructed respectively, and distinct discrimination was obtained for each comparison (**Supplementary Figure S9**). To further investigate the different interventional effects of XBJ and BIPM in alone or in combination, we explored the metabolic phenotypes of different groups focused on the level changes of identified potential biomarkers. In total, 36 metabolites were reversely regulated by XBJ when compared with Model group, mainly including glycerophospholipids, sphingolipids, FFAs, bile acids and acylcarnitines; 42 metabolites were regulated by BIPM, mainly including amino acids, glycerophospholipids, and acylcarnitines; 72 metabolites were regulated after XBJ-BIPM combination treatment, including most of the 91 potential biomarkers. Detailed information of the metabolites was presented in **Table 1**. It was shown that the combination intervention induced more altered metabolites, 15 metabolites of which were not affected when XBJ or BIPM was administrated alone. A venn diagram was described to display the overlapping relationship of regulated metabolites for different comparisons (**Figure 2A**), and the classification of regulated metabolites for different comparisons was shown in **Figure 2B**.

Compared with Model group, there were no significant changes in bile acids profile (ursodeoxycholic acid, deoxycholic acid, and chenodeoxyglycocholic acid), creatinine, uric acid, and allantoin in BIPM group, but the levels of these metabolites were apparently down-regulated by the combination of BIPM and XBJ. The results evidently showed that treatment with this combination conferred protection against liver and kidney dysfunctions caused by infection in sepsis. Similarly, the decreased levels of FFAs in COM group compared with Model group might be the defensive consequence of XBJ against inflammation and oxidative stress. Beyond this point, more amino acids, glycerophospholipids, and acylcarnitines were regulated after combination treatment compared with XBJ or BIPM monotherapy, exhibiting a synergistic performance of XBJ and BIPM as well.

**Figure 3:**
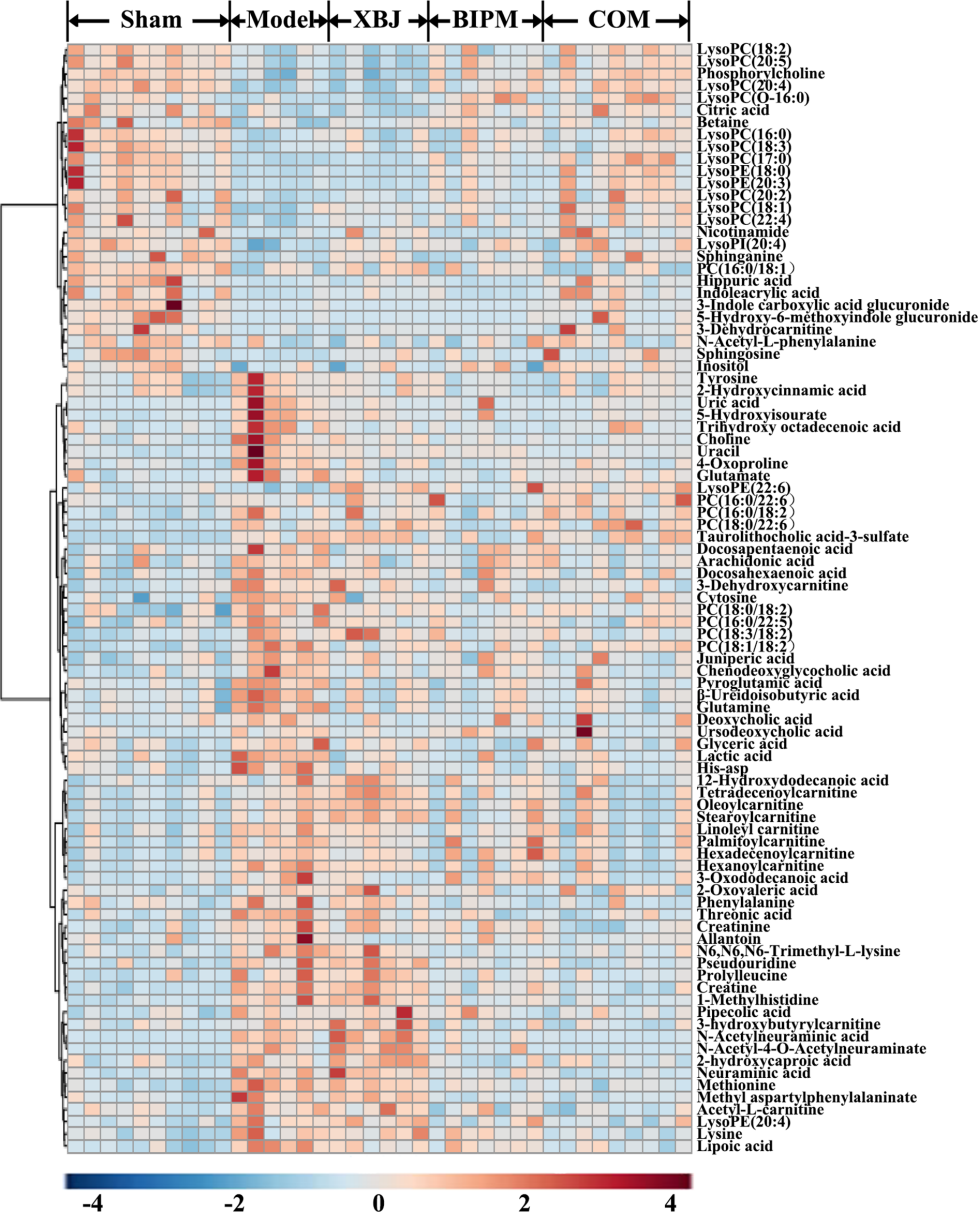
Heat map showing the level changing trend of 91 differential metabolites across the five groups. The colors from blue to red indicate higher levels of metabolites. XBJ, xuebijing injection; BIPM, biapenem; COM, combination of xuebijing injection and biapenem.

**Figure 4:**
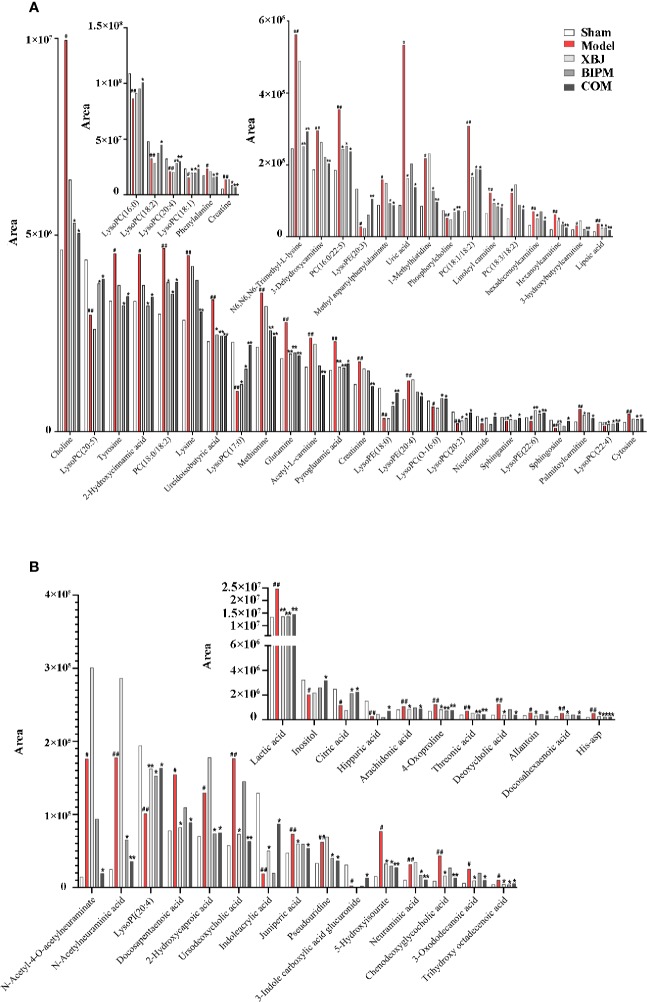
The average peak areas of each metabolite in each group for the 72 metabolites detected in positive **(A)** and negative **(B)** ion modes. ^#^*p* < 0.05, ^##^*p* < 0.01 versus the Sham group; ^*^p < 0.05, ^**^p < 0.01 versus the Model group. XBJ, xuebijing injection; BIPM, biapenem; COM, combination of xuebijing injection and biapenem.

Furthermore, a heat map was depicted to intuitively show the content changing trend of 91 identified potential biomarkers among different groups. As shown in **Figure 3**, an obvious color discrimination was observed between Model group and Sham group, and different metabolites were reversibly regulated in XBJ, BIPM, and COM group respectively. The COM and Sham group shared similar color distribution, indicating an excellent synergistic effect of XBJ-BIPM combination for sepsis. Among these 91 metabolites, we focused on 72 metabolites which could be reversely regulated by XBJ-BIPM combination, and the average peak area of each metabolite in each group was shown in **Figure 4**. The histograms directly exhibited levels of 72 metabolites across the five groups.

To explore the latent relationships of potential biomarkers, a heat map was produced according to the Pearson correlation coefficients between metabolites (**Supplementary Figure S10**). The levels of LysoPCs and LysoPEs showed an obvious negative correlations with FFAs, bile acids, and acylcarnitines, while acylcarnitines had significant positive correlation with part of FFAs and amino acids.

### Metabolic Pathway Analysis

**Figure 5:**
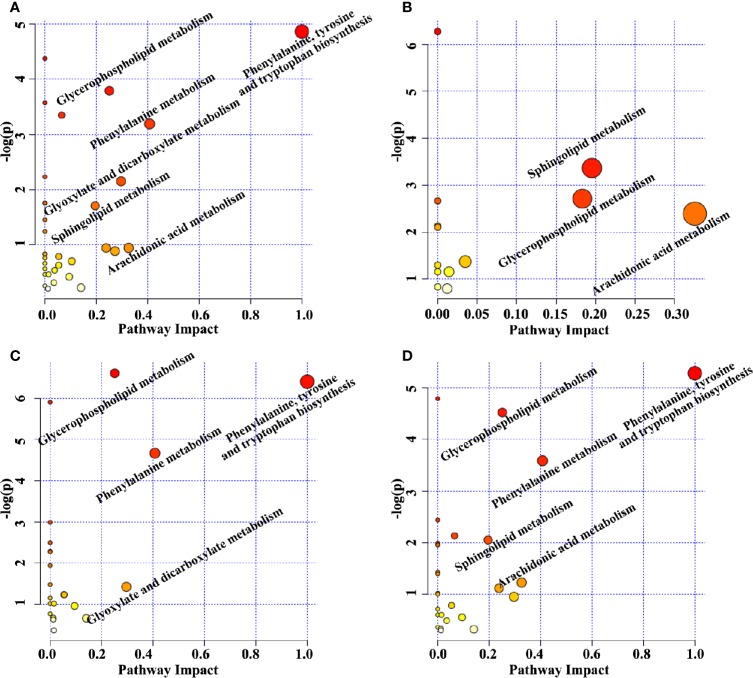
Topology maps of the altered metabolic pathways analyzed by metaboanalyst between Model group *vs.* Sham group **(A)**, XBJ group *vs.* Model group **(B)**, BIPM group *vs.* Model group **(C)**, COM group *vs.* Model group **(D)**. XBJ, xuebijing injection; BIPM, biapenem; COM, combination of xuebijing injection and biapenem.

**Figure 6:**
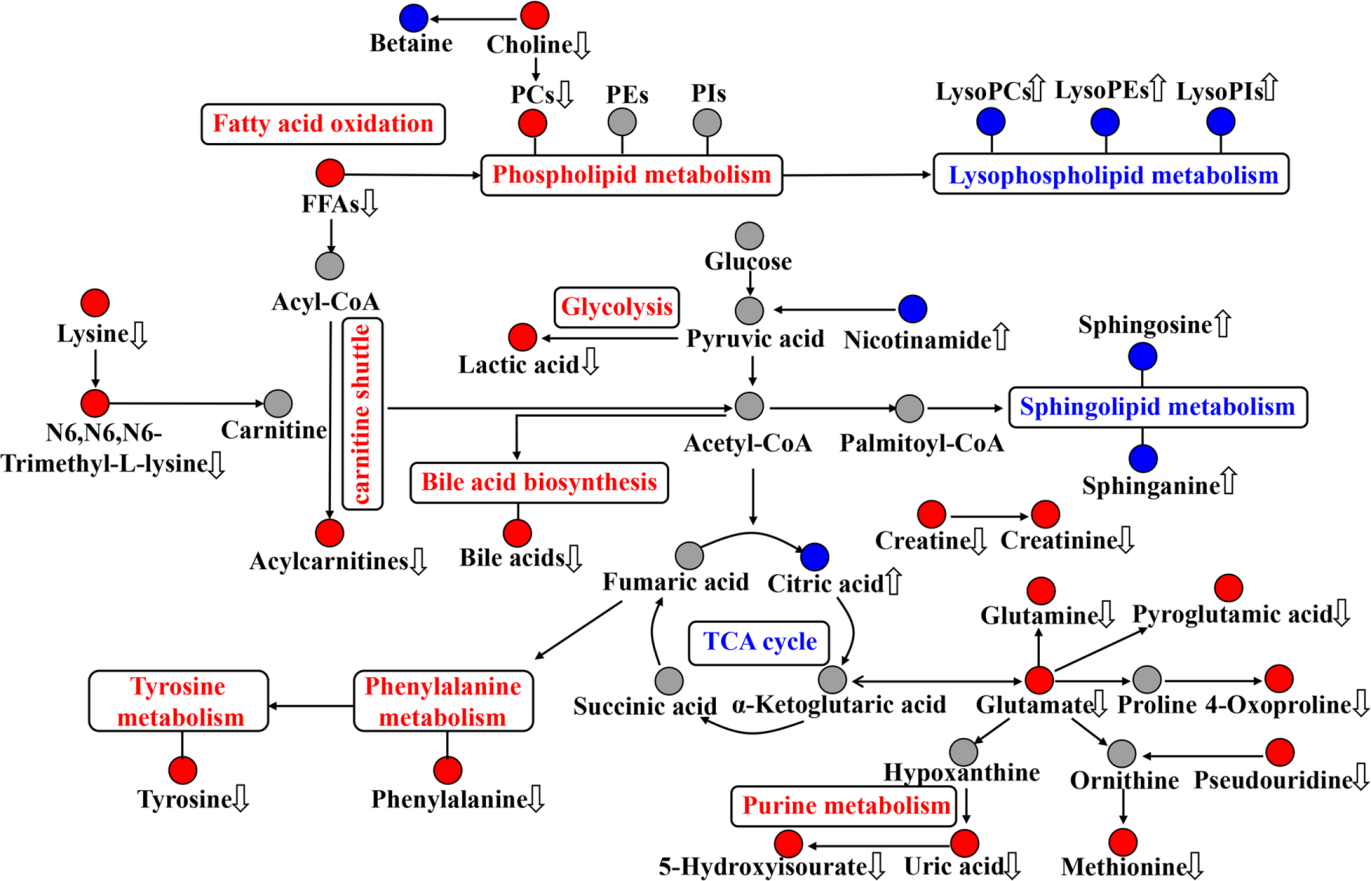
Disordered metabolic pathway networks in sepsis and the interventional effects of biapenem (BIPM) in combination with xuebijing injection (XBJ). The red dots represent increased metabolites in septic rats, the blue dots represent decreased metabolites in septic rats, and the gray dots represent undetected metabolites. The disturbed metabolic pathways in septic rats are marked in red (up-regulated) and blue (down-regulated). The metabolites regulated by the combination of XBJ and BIPM are marked with up and down arrows.

The perturbed metabolic pathways were enriched on the basis of altered metabolites. The principle disturbed metabolic pathways for Model group included phenylalanine, tyrosine and tryptophan biosynthesis, glycerophospholipid metabolism, phenylalanine metabolism, glyoxylate and dicarboxylate metabolism, sphingolipid metabolism, and arachidonic acid metabolism (**Figure 5A**), which were valuable for elucidating pathogenic mechanism of sepsis. Compared with Model group, 13, 22, and 27 metabolic pathways were regulated by XBJ, BIPM, and XBJ-BIPM combination, respectively (**Figures 5B–D**). The results suggested that many more metabolic pathways were affected by the synergistic treatment of XBJ and BIPM. A metabolic correlation network (**Figure 6**) was further established by searching online database KEGG (https://www.kegg.jp/) and HMDB (http://www.hmdb.ca/).

## Discussion

In this work, we characterized the metabolic phenotype of sepsis and described metabolic footprints change of septic rats responding to XBJ and BIPM individually and in combination, in addition to histopathological and survival evaluation. This study was the first to employ metabolomics to elucidate the synergistic effect and decipher the underlying mechanisms of BIPM in combination with XBJ against sepsis. The major findings on the synergistic effect of this combination in our study include: 1) BIPM monotherapy regulated metabolism levels of amino acids, glycerophospholipids, and acylcarnitines in sepsis, but had no effect on sphingolipids, bile acids and FFAs. 2) Many more endogenous metabolites and metabolic pathways were significantly regulated after combination treatment compared with XBJ or BIPM monotherapy. 3) Metabolisms of lipids, amino acids, acylcarnitines, and bile acids were common pathways involved in the synergistic action of XBJ and BIPM.

### Lipid Metabolism

The perturbed lipid metabolism including reduced lysophospholipids (LPLs) metabolism, increased PCs metabolism, elevated FFAs metabolism, and decreased sphingolipids metabolism as reflective of systemic changes resulted from inflammatory response and oxidative stress in sepsis (Sevastou et al., 2013). Our results were consistent with previous studies (Mecatti et al., 2018). Decreased LysoPCs might enhance the production of lysophosphatidic acids (Tokumura, 2002), which induced an immune response by activating various immune cells (Gräler and Goetzl, 2002). The LysoPCs have been considered useful for discriminating septic patients from normal control (Drobnik et al., 2003; Cho et al., 2012), and another research demonstrated that LysoPCs increased after administration with appropriate antibiotics in septic patients (Park et al., 2014), which was also agreement with our results. Elevated PCs and reduced LysoPCs might be related to a lack of phospholipase A2 (PLA2), which could hydrolyze PCs to LysoPCs. After intervention by XBJ, BIPM and XBJ-BIPM combination, a total of 8, 12, and 17 glycerophospholipids were reversely regulated respectively, and PLAs probably acted as the therapeutic targets. In addition, lipolysis, an adaptive response to inflammation (Fong et al., 1990), together with increased biosynthesis and decreased oxidation in liver, leading to the accumulation of FFAs in plasma of septic rats (Kamisoglu et al., 2013). The increased arachidonic acid, a precursor of pro-inflammation eicosanoid mediators (Sanak, 2016), might be closely related to the systemic inflammation reaction in sepsis. Sphingolipids are important building blocks of cell membranes, and a large of evidences have demonstrated that sphingolipid metabolites participate in many cell regulation processes as key signal molecules in immunity and inflammation (Michael and Sarah, 2014). Previous studies had shown that immune response in sepsis was associated with disordered sphingolipid metabolism (Winkler et al., 2015). In our study, FFAs and sphingolipids could barely be affected by BIPM monotherapy, but they were well regulated when BIPM combined with XBJ, indicating a synergistic effect of this combination. In short, BIPM in combination with XBJ resulted in enhanced regulating effect on perturbed lipid metabolism in sepsis.

### Amino Acid Metabolism

The alteration of amino acid metabolism was another metabolic characteristic of sepsis. Our results showed that both BIPM used alone and combined with XBJ displayed excellent effect on the regulation of amino acids in septic rats, but XBJ monotherapy had a weaker action on amino acids. It was observed that most amino acids elevated in sepsis compared with Sham group, and this results seemed contrary with our previous report (Zuo et al., 2018). In fact, it should be noticed that the time of blood sampling in this work was much later than that of our previous work. In the early stage of sepsis, the organism was in a state of hypermetabolism, and peripheral protein catabolism was activated (Su et al., 2015). Augmented metabolic activity in the liver region promoted the shift of amino acids from peripheral tissues to liver for the hepatic protein synthesis (Druml et al., 2001). However, as the disease progressed, this protein synthesis was reduced in liver because of the hepatic dysfunction (Vaidyanath et al., 1976). Increased muscle protein breakdown and relative hepatic incompetence resulted in accumulation of amino acids (Freund et al., 1979). These reasons might explain the opposite trend of plasma amino acids in our previous study and this work. In this aspect, the plasma accumulation of aromatic and sulfur containing amino acids (phenylalanine, tyrosine, and methionine), which must be metabolized by the liver, was likely to be an important indicator of the combined effect of muscle breakdown and hepatic dysfunction in sepsis.

### Energy Metabolism

Elevated levels of acylcarnitines suggested activated fatty acids oxidation (FAO) and energy metabolism in sepsis. The carnitine shuttle, as an essential pathway to transport fatty acids from cytoplasm to mitochondria, played a pivotal role in β-oxidation (Adeva-Andany et al., 2017). Long-chain acyl-CoAs were converted to acylcarnitines by carnitine acyl transferase in the process (Calvani et al., 2000). As one of the major ways of energy production, FAO enhanced in septic rats to maintain the energy supplement, resulting in the increased acylcarnitines. In this work, more acylcarnitines were down-regulated after combination treatment compared with XBJ or BIPM monotherapy. The results implied that energy metabolism was alleviated under the synergistic effect of XBJ and BIPM.

### Bile Acid Metabolism

Septic patients usually exhibit cholestasis, and plasma bile acid levels have been viewed as related to mortality (Thomas, 2017). In the present study, elevated bile acids were observed in septic rats, which implied a liver dysfunction induced by infection. It has been suggested that bile acids accumulation promoted sepsis-associated inflammation *via* NLRP3 inflammasome activation (Hao et al., 2017). The bile acids were decreased after administration of XBJ, but not regulated by BIPM. This phenomenon might attribute to multiple components with hepatoprotective activities existed in XBJ. Therefore, the combination would have more advantages against sepsis in clinic.

In addition, plasma lactic acid levels are traditionally interpreted as a marker of tissue hypoxia and usually employed as an indicator of severity and outcome of sepsis in clinical practice (Nolt et al., 2018). A significant increase of lactic acid in septic rats was also observed in this work, and it was reversely regulated by BIPM in combination with XBJ. The results indicated that administration of this combination could improve hypoxia and energy metabolism in sepsis. Uric acid is a product of purine metabolism and considered as an independent risk factor for weakness in kidney function (Pilemann-Lyberg et al., 2019). In most mammals, uric acid is further oxidized by the enzyme uricase into allantoin (Andries et al., 2018). Creatinine is a biomarker for renal function in clinic. Therefore, the elevated levels of uric acid, allantoin, and creatinine demonstrated kidney dysfunction in sepsis. These metabolites were down-regulated by XBJ-BIPM combination, suggesting a renal protection of this combination.

To sum up, we described a UHPLC-Q-Orbitrap HRMS method in both positive and negative modes for metabolomic evaluation of synergistic effect of BIPM in combination with XBJ against sepsis. The two drugs may interact each other to regulate the plasma levels of amino acids, glycerophospholipids, sphingolipids, FFAs, acylcarnitines, and bile acids in sepsis. The specific effects of these endogenous metabolites in sepsis therapy and metabolomics-based key pathways remain to be further researched. Nevertheless, this work provided a better understanding on the synergistic mechanisms of XBJ and BIPM from metabolomic insights. The results provide some support for clinical application of antibiotics in combination with XBJ and have important implications for the treatment of sepsis in clinic.

## Data Availability Statement

The datasets generated for this study are available on request to the corresponding authors.

## Ethics Statement

The animal study was reviewed and approved by the Animal Ethics Committee of the first affiliated hospital of Zhengzhou University.

## Author Contributions

L-WL and Y-YS contributed equally to this work. ZS and X-JZ designed the research. L-WL, Y-YS, Z-LL, and L-HZ performed the experiments. L-WL, Y-YS, MT, and Z-WJ analyzed data. L-WL and H-YZ wrote the manuscript. PX, LZ, and Q-ZD revised the manuscript. All authors read and approved the final manuscript.

## Funding

This study was supported by the National Natural Science Foundation of China (81873188, 81703666), the Key Research and Promotion Project of Henan Province (182102310243), the Key Scientific Research Project of Colleges and Universities in Henan Province (20A350018, 18A360022), and Hong Ri Medical Research Foundation (HRJJ18002).

## Conflict of Interest

The authors declare that the research was conducted in the absence of any commercial or financial relationships that could be construed as a potential conflict of interest.
