# Supplementary material for: Metabolomic Insights Into the Synergistic Effect of Biapenem in Combination With Xuebijing Injection Against Sepsis
Source: Front Pharmacol. 2020 Apr 22;11:502. doi: 10.3389/fphar.2020.00502 (PMC7189733; doi:10.3389/fphar.2020.00502)
Supplement: Supplementary file 1 [file DataSheet_1.pdf]

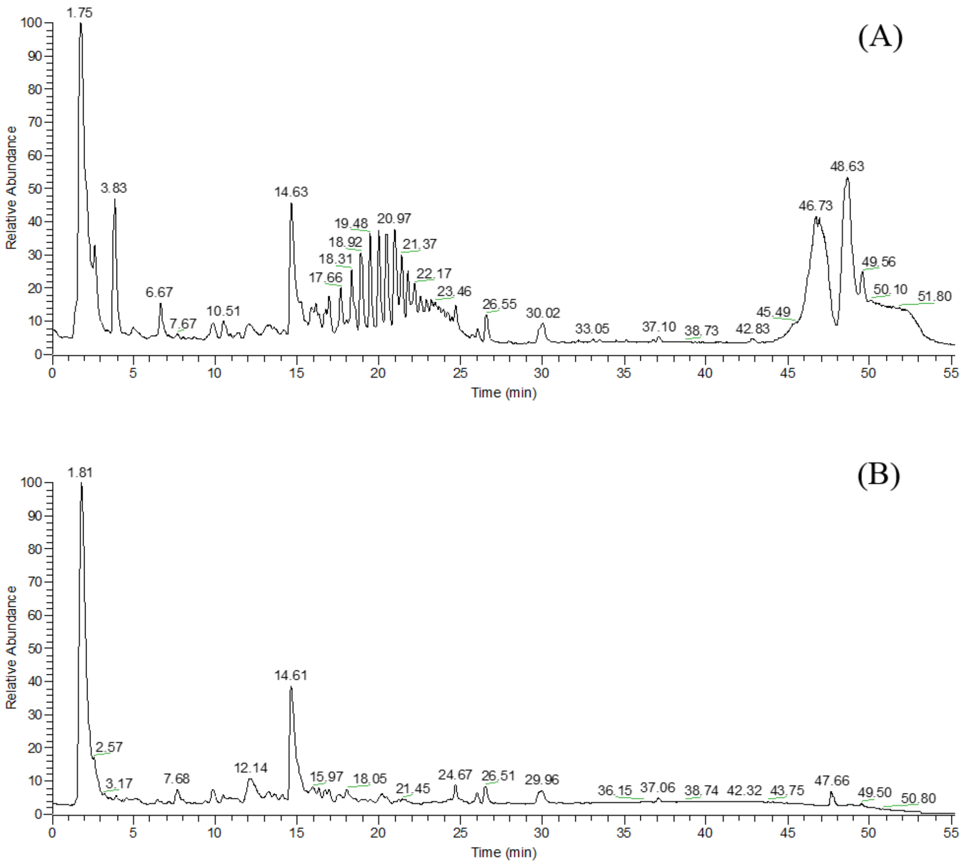

Supplementary Figure S1. The total ion chromatograms of xuebijing injection in positive ion mode **(A)** and negative ion mode **(B)**.

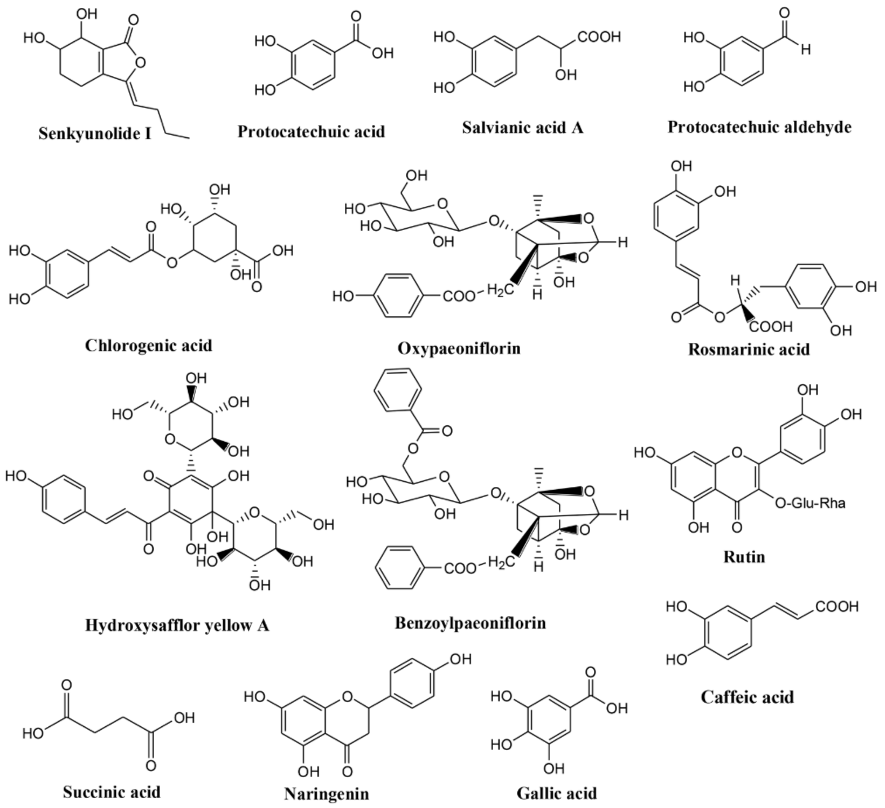

Supplementary Figure S2. Chemical structures of 14 active components contained in xuebijing injection.

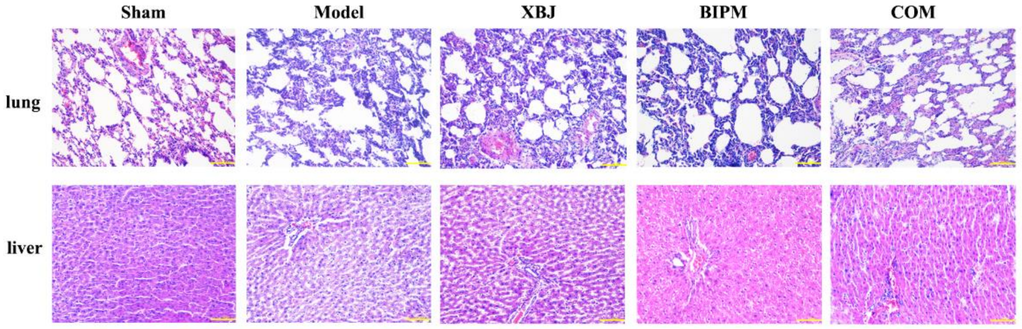

Supplementary Figure S3. Effect of XBJ, BIPM, and XBJ-BIPM combination on the histological morphology of lung and liver tissues in septic rats (HE staining, ×100). XBJ, xuebijing injection; BIPM, biapenem; COM, combination of xuebijing injection and biapenem.

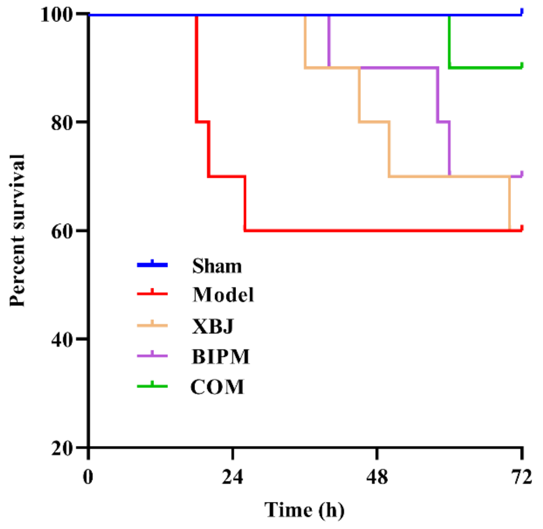

Supplementary Figure S4. Survival curves of rats in Sham, Model, XBJ, BIPM, and COM groups. XBJ, xuebijing injection; BIPM, biapenem; COM, combination of xuebijing injection and biapenem.

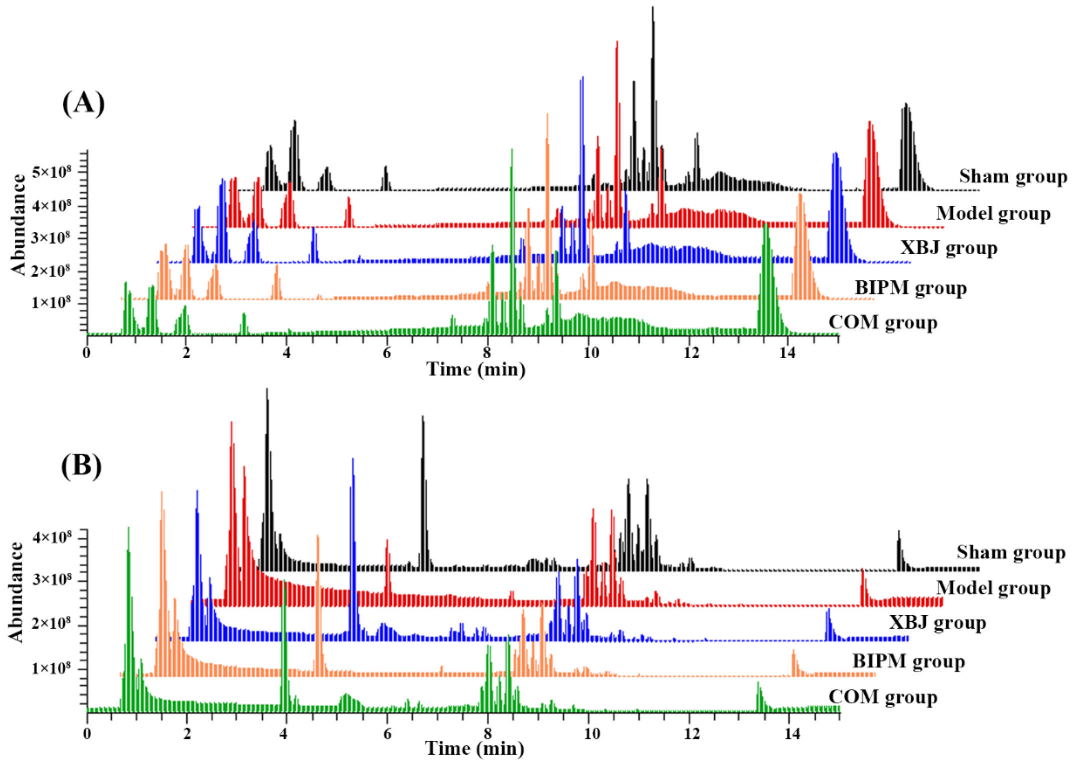

Supplementary Figure S5. Representative base peak chromatograms of plasma samples from five groups detected in both positive **(A)** and negative **(B)** ion modes.

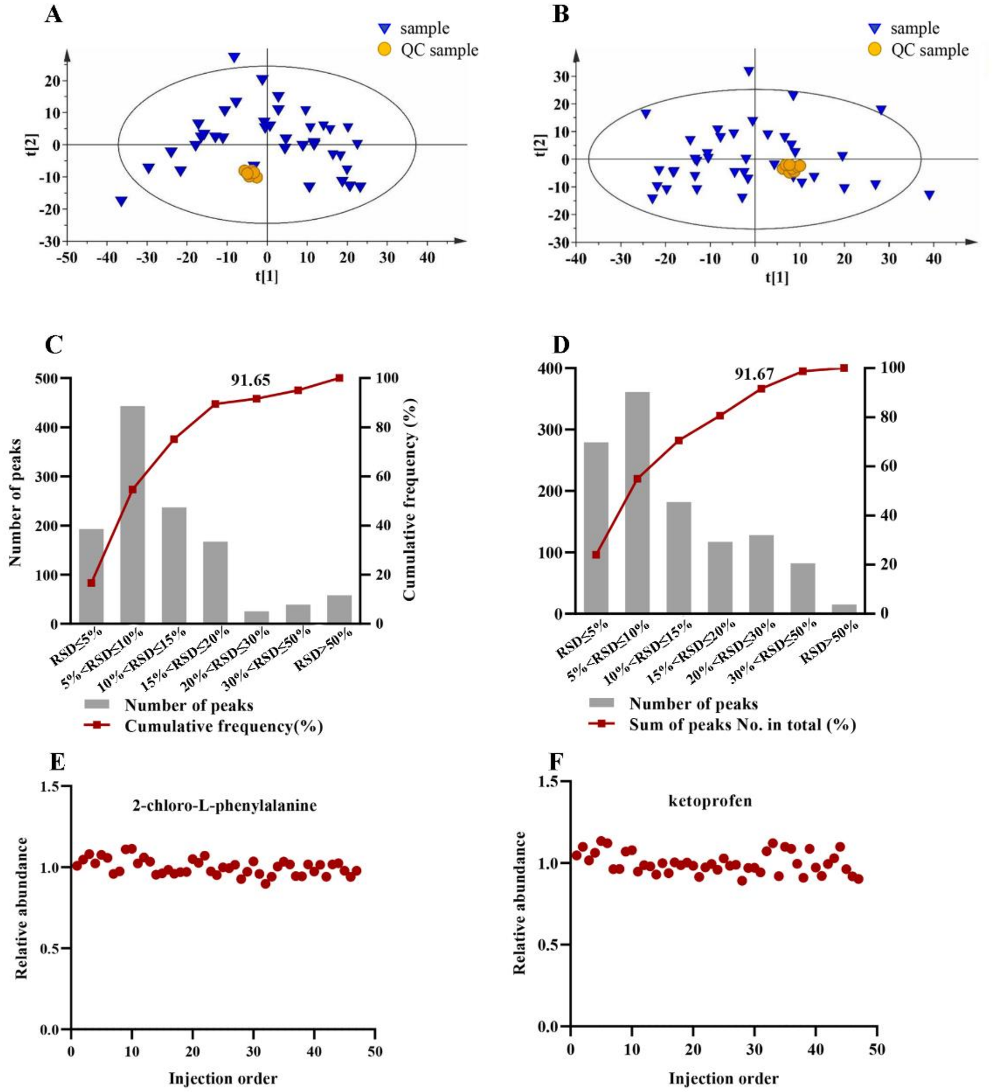

Supplementary Figure S6. Reproducibility and stability assessment of the analytical strategy by the performance of QC samples and responses of internal standard for both positive and negative ion modes. PCA score plot in positive **(A)** and negative **(B)** ion modes; Distribution of relative standard deviation (RSD) for metabolic ion features of QC samples detected in positive **(C)** and negative **(D)** ion modes; The relative abundance variabilities with respect to injection order for internal standards in positive **(E)** and negative **(F)** ion modes. QC, quality control; PCA, principal component analysis.

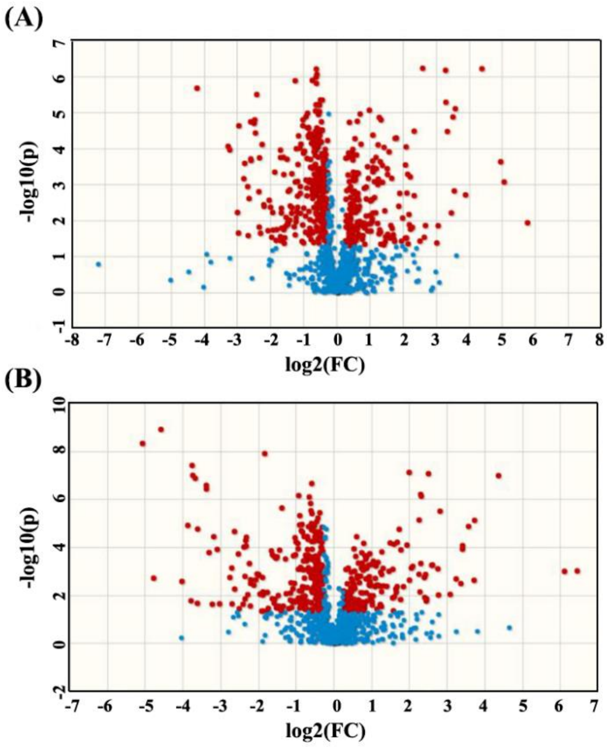

Supplementary Figure S7. The volcano plot of metabolites for the comparison between Model group *vs.* Sham group in positive **(A)** and negative **(B)** ion modes. The red dots represented metabolites with *p* values <0.05 and fold change values >1.20 (or <0.83).

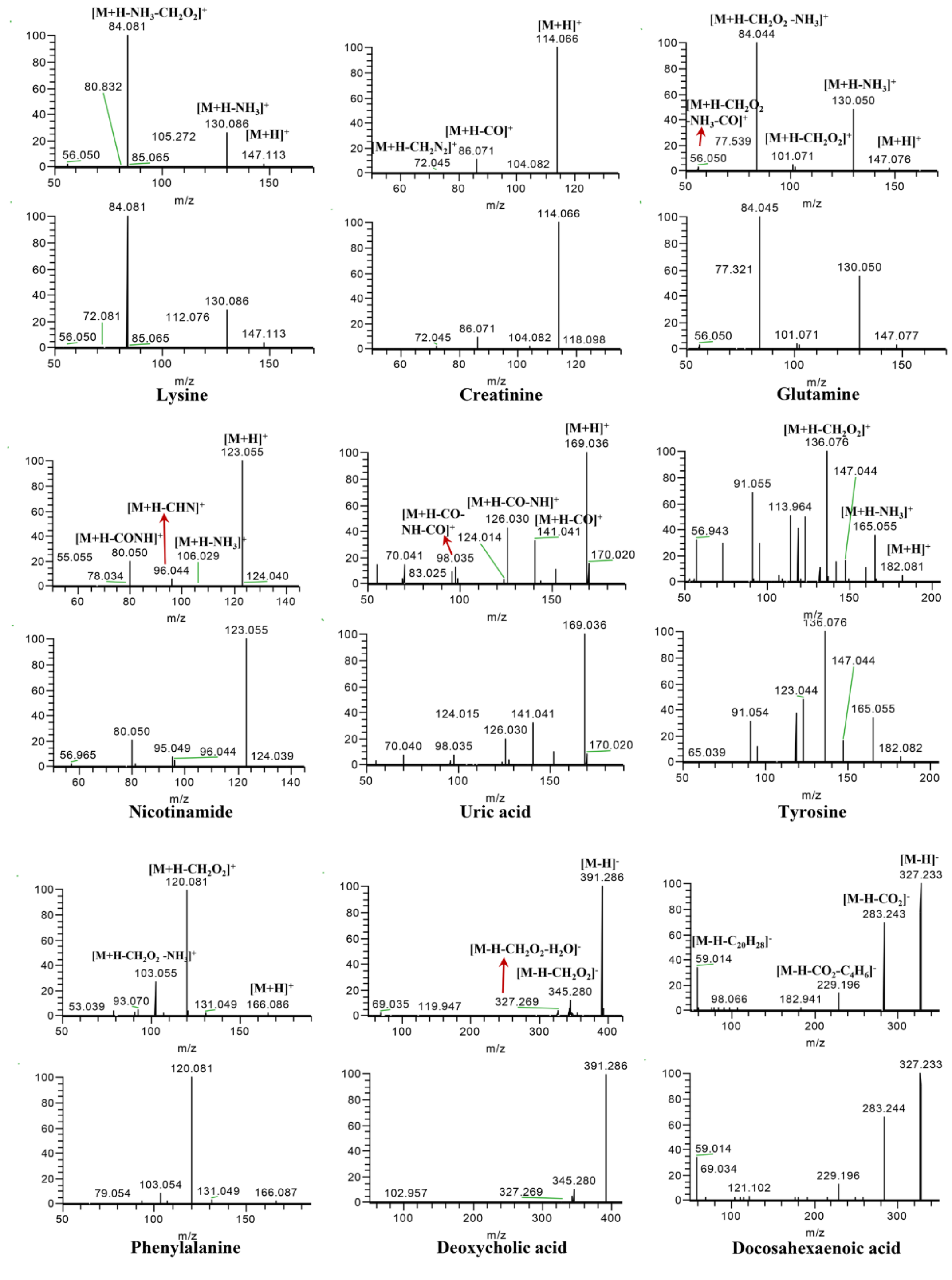

Supplementary Figure S8. The MS/MS spectra and fragment ions for some of these metabolites in certain samples compared with standard references.

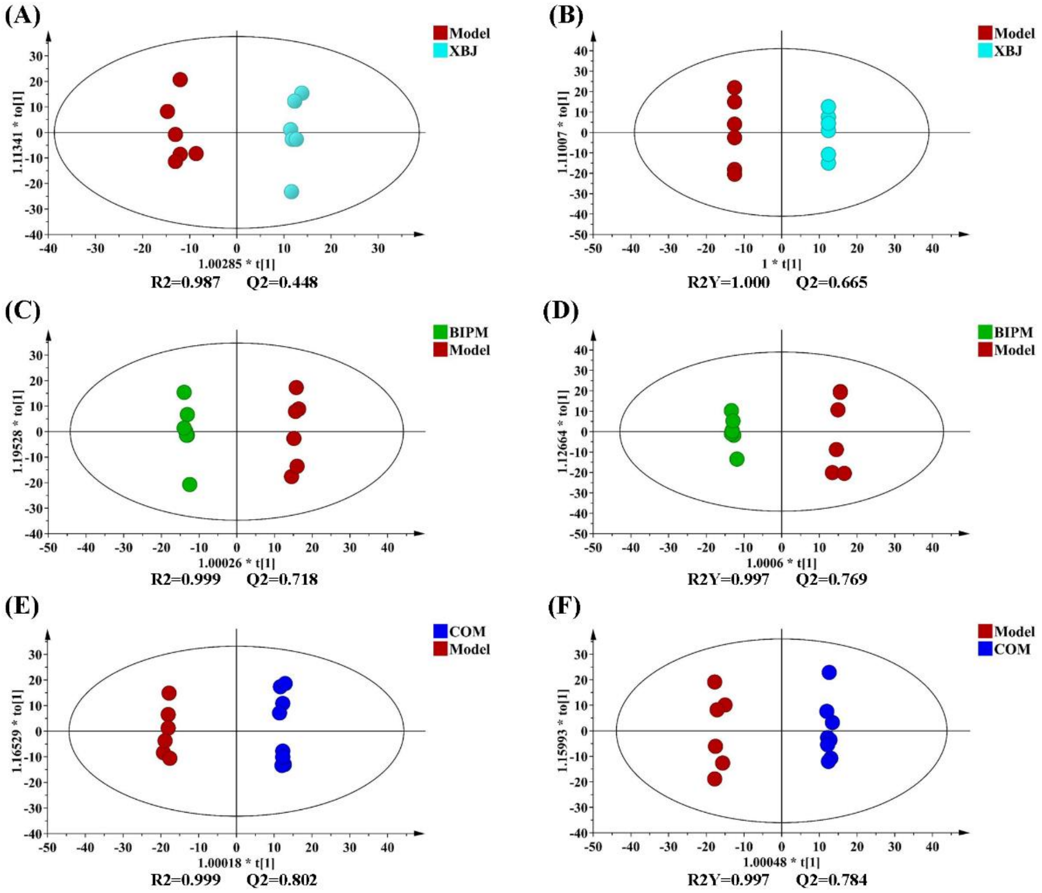

Supplementary Figure S9. OPLS-DA score plots for XBJ *vs*. Model, BIPM *vs*. Model, and COM *vs*. Model in positive **(A, C, E, G)** and negative **(B, D, F, H)** ion modes. XBJ, xuebijing injection; BIPM, biapenem; COM, combination of xuebijing injection and biapenem. OPLS-DA, orthogonal partial least-squares discriminant analysis.

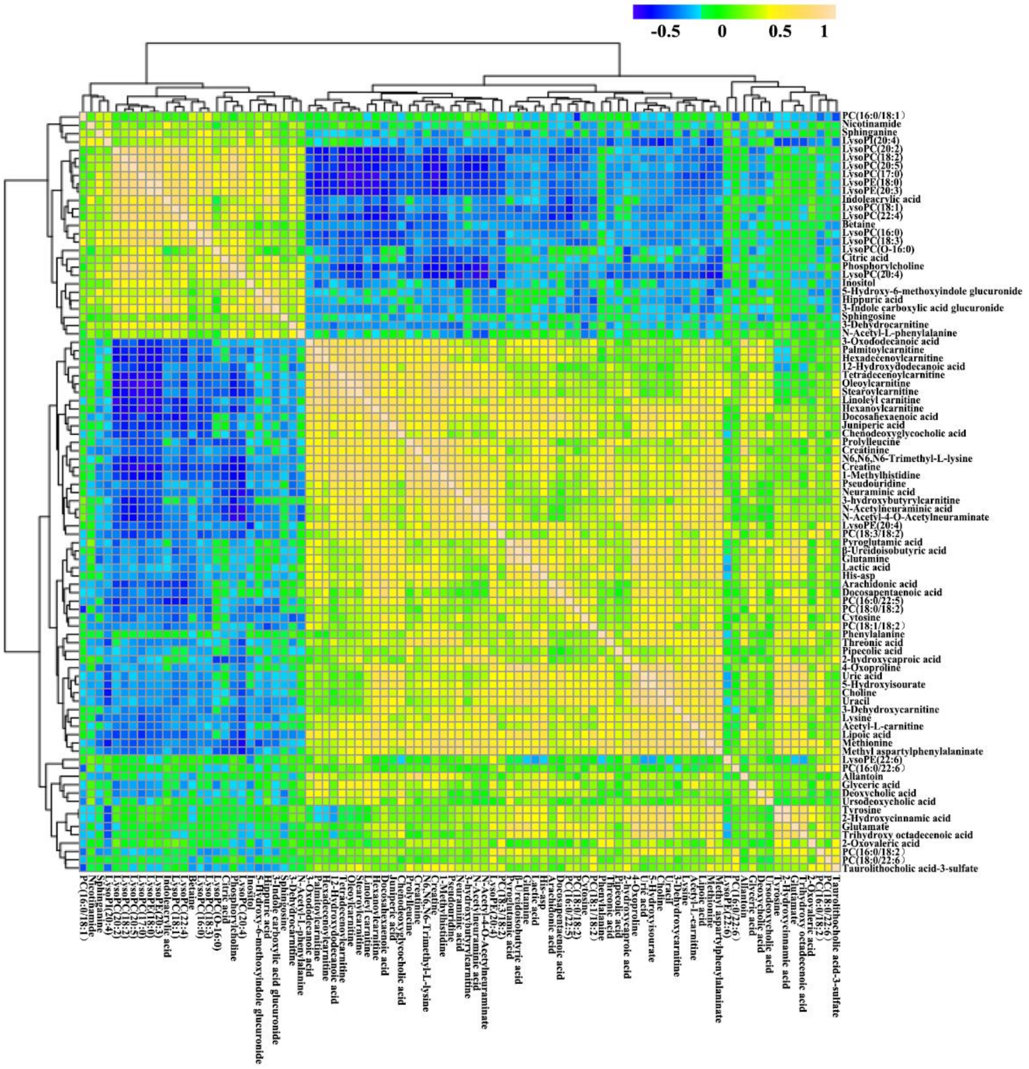

Supplementary Figure S10. Heat map of the Pearson correlation coefficients calculated between the 91 differential metabolites in different groups.
