# Supplementary material for: Metabolomic Insights Into the Synergistic Effect of Biapenem in Combination With Xuebijing Injection Against Sepsis
Source: Front Pharmacol. 2020 Apr 22;11:502. doi: 10.3389/fphar.2020.00502 (PMC7189733; doi:10.3389/fphar.2020.00502)
Supplement: Supplementary file 2 [file Table_1.docx]

**Supplementary Table S1** Plant names of five herbs in xuebijing injection.

|  | **Plant common name** | **Plant full scientific name**  **Kew MPNS** | **Plant full scientific name**  **Chinese Pharmacopoeia** |
| --- | --- | --- | --- |
| 1 | Honghua | Carthamus tinctorius L. | Carthami Flos |
| 2 | Chishao | Paeonia lactiflora Pall. | Paeoniae Radix Rubra |
| 3 | Chuanxiong | Conioselinum anthriscoides 'Chuanxiong' | Chuanxiong Rhizoma |
| 4 | Danshen | Salvia miltiorrhiza Bunge | Salviae Miltiorrhizae Radix et Rhizoma |
| 5 | Danggui | Angelica sinensis (Oliv.) Diels | Angelicae Sinensis Radix |
