# Supplementary material for: Metabolomic Insights Into the Synergistic Effect of Biapenem in Combination With Xuebijing Injection Against Sepsis
Source: Front Pharmacol. 2020 Apr 22;11:502. doi: 10.3389/fphar.2020.00502 (PMC7189733; doi:10.3389/fphar.2020.00502)
Supplement: Supplementary file 3 [file Table_2.docx]

**Supplementary Table S2** The identified chemical ingredients of xuebijing injection.

| **No.** | **Compounds** | **t_R_ (min)** | **Formula** | **Ion mode** | **ES/expected  (m/z)** | **ES/measured**  **(m/z)** | **Delta  (ppm)** | **Fragment Ions (m/z)** |
| --- | --- | --- | --- | --- | --- | --- | --- | --- |
| 1 | 5-Hydroxymethylfurfural^*^ | 1.68 | C_6_H_6_O_3_ | N | 125.02441 | 125.02387 | -4.378 | 97.03[M-H-CO]-, 69.03[M-H-CO-CO]- |
| 2 | Succinic acid^*^ | 2.49 | C_4_H_6_O_4_ | N | 117.01933 | 117.01897 | -3.093 | 99.01[M-H-H2O]-,73.03[M-H-CO2]- |
| 3 | Gallic acid^*^ | 2.86 | C_7_H_6_O_5_ | N | 169.01424 | 169.01390 | -2.050 | 125.02[M-H-CO2]- |
| 4 | 1’-O-Galloylsucrose | 2.99 | C_19_H_26_O_15_ | N | 493.11989 | 493.11905 | -1.710 | 313.06[M-H-C6H12O6]-,169.01[M-H-C6H12O6-C6H8O4],125.02[M-H-C6H12O6-C6H8O4-CO2]- |
| 5 | 6’-O-galloylsucrose isomer | 2.99 | C_19_H_26_O_15_ | N | 493.11989 | 493.11905 | -1.710 | 313.06[M-H-C6H12O6]-,169.01[M-H-C6H12O6-C6H8O4],125.02[M-H-C6H12O6-C6H8O4-CO2]-, |
| 6 | 6’-O-galloylsucrose isomer | 2.99 | C_19_H_26_O_15_ | N | 493.11989 | 493.11905 | -1.710 | 313.06[M-H-C6H12O6]-,169.01[M-H-C6H12O6-C6H8O4],125.02[M-H-C6H12O6-C6H8O4-CO2]-, |
| 7 | Matrine | 3.38 | C_15_H_24_ON_2_ | P | 249.19613 | 249.19548 | -2.648 | 176.11[M-H-C4H8-NH3]-,148.11[M-H-C4H8-NH3-CO]-,120.08[M-H-C4H8-NH3-CO-C2H4]-,110.10[M-H-C4H8-NH3-CO-C3H2]-,98.06[M-H-C4H8-NH3-C6H6]-,84.08[M-H-C4H8-NH3-CO-C5H4]-,56.05[M-H-C4H8-NH3-CO-C7H8]- |
| 8 | Vanillic acid isomer | 4.34 | C_8_H_8_O_4_ | N | 167.03498 | 167.03464 | -2.048 | 149.02[M-H-H2O]-,139.04[M-H-CO]-,123.04[M-H-CO2]-,109.03[M-H-CO2-CH2]- |
| 9 | Vanillic acid isomer | 4.34 | C_8_H_8_O_4_ | N | 167.03498 | 167.03464 | -2.048 | 123.04[M-H-CO2]- |
| 10 | Ethyl gallate isomer | 4.54 | C_9_H_10_O_5_ | N | 197.04554 | 197.04517 | -1.912 | 179.03[M-H-H2O]-,135.04[M-H-H2O-CO2]-,123.04[M-H-H2O-CO-CO]-,109.03[M-H-H2O-CO-CO-CH2]- |
| 11 | Salvianic acid A^*^ | 4.54 | C_9_H_10_O_5_ | N | 197.04554 | 197.04517 | -1.912 | 179.03[M-H-H2O]-,151.04[M-H-H2O-CO]-,135.04[M-H-H2O-CO2]-,123.04[M-H-H2O-CO-CO]-,109.03[M-H-H2O-CO2-C2H2]- |
| 12 | Protocatechuic acid^*^ | 5.12 | C_7_H_6_O_4_ | N | 153.01933 | 153.01898 | -2.300 | 109.03[M-H-CO2]-,91.02[M-H-CO2-H2O]-,81.03[M-H-CO2-CO]- |
| 13 | Paeonol | 5.12 | C_9_H_10_O_3_ | P | 167.07027 | 167.07021 | -0.363 | 152.05[M+H-CH3]+,123.04[M+H-C2H4O]+,95.05[M+H-C2H4O-CO]+ |
| 14 | Methyl gallate | 6.36 | C_8_H_8_O_5_ | N | 183.02989 | 183.02945 | -2.44 | 168.01[M-H-CH3]-,139.04[M-H-CO2]-,124.02[M-H-CO2-CH3]- |
| 15 | p-Anisic acid isomer | 6.43 | C_8_H_8_O_3_ | N | 151.04006 | 151.03975 | -2.101 | 133.03 [M-H-H2O]-,123.05[M-H-CO]-,107.05[M-H-CO2]-,93.03[M-H-CO2-CH2]-, |
| 16 | 6’-O-galloyl-Desbenzoylpaeoniflorin Isomer | 7.05 | C_23_H_28_O_14_ | N | 527.14062 | 527.13995 | -1.287 | 497.13[M-H-CH2O]-,399.09[M-H-C6H8O3]-,313.06[M-H-C10H14O5]-,271.05[M-H-C10H14O5-C2H2O]-,211.02[M-H-C10H14O5-C2H2O-C2H4O2]-,169.01[M-H-C10H14O5-C6H8O4]-,125.02[M-H-C10H14O5-C6H8O4-CO2]- |
| 17 | 4-O-Methyldesbenzoylpaeoniflorin | 7.16 | C_17_H_26_O_10_ | N | 389.14532 | 389.14496 | -0.925 | 343.14[M-H-CH2O2]-,181.09[M-H-CH2O2-C6H10O5]-,166.06[M-H-CH2O2-C6H10O5-CH3]-,163.08[M-H-CH2O2-C6H10O5-H2O]-,161.05[M-H-CH2O2-C10H14O3]-,151.08[M-H-CH2O2-C6H10O5-CH2O]-,136.05[M-H-CH2O2-C6H10O5-CH2O-CH3]-,109.07[M-H-CH2O2-C6H10O5-CH2O-C2H2O]- |
| 18 | Mudanpioside F | 7.16 | C_16_H_24_O_8_ | N | 343.13984 | 343.13922 | -1.809 | 181.09[M-H-C6H10O5]-,151.08[M-H-C6H10O5-CH2O]-,109.07[M-H-C6H10O5-CH2O-C2H2O]- |
| 19 | Protocatechuic aldehyde isomer | 7.66 | C_7_H_6_O_3_ | N | 137.02441 | 137.02402 | -2.900 | 93.03[M-H-CO2]- |
| 20 | Protocatechuic aldehyde isomer | 7.66 | C_7_H_6_O_3_ | N | 137.02441 | 137.02402 | -2.900 | 109.03[M-H-CO],93.03[M-H-CO2]-, |
| 21 | Protocatechuic aldehyde isomer | 7.66 | C_7_H_6_O_3_ | N | 137.02441 | 137.02402 | -2.900 | 93.03[M-H-CO]-, |
| 22 | Protocatechuic aldehyde^*^ | 7.66 | C_7_H_6_O_3_ | N | 137.02441 | 137.02402 | -2.900 | 93.03[M-H-CO2]- |
| 23 | 6’-O-galloyl-Desbenzoylpaeoniflorin Isomer | 8.74 | C_23_H_28_O_14_ | N | 527.14062 | 527.13971 | -1.743 | 313.06[M-H-C10H14O5]-,271.05[M-H-C10H14O5-C2H2O]-,211.02[M-H-C10H14O5-C2H2O-C2H4O2]-,169.01[M-H-C10H14O5-C6H8O4]-,125.02[M-H-C10H14O5-C6H8O4-CO2]- |
| 24 | Catechin^*^ | 9.39 | C_15_H_14_O_6_ | N | 289.07176 | 289.07135 | -1.423 | 245.08[M-H-CO2]-,221.08[M-H-CO-CO-C]-,203.07[M-H-CO-CO-CH2O]-,179.03[M-H-C6H6O2]-,165.02[M-H-C6H6O2-CH2]-,151.04[M-H-C6H6O2-CO]-,137.02[M-H-C6H6O2-CO-CH2],125.02[M-H-C6H6O2-CO-C2H2]-,109.03[M-H-C9H8O4]- |
| 25 | Oxypaeoniflorin^*^ | 9.84 | C_23_H_28_O_12_ | N | 495.15079 | 495.15012 | -1.372 | 333.10[M-H-C6H10O5]-,195.07[M-H-C6H10O5-C7H6O3]-,137.02[M-H-C6H10O5-C10H12O4]-,93.03[M-H-C6H10O5-C10H12O4-CO2]- |
| 26 | Oxypaeoniflorin isomer | 9.91 | C_23_H_28_O_12_ | N | 495.15079 | 495.15009 | -1.433 | 465.14[M-H-CH2O]-,165.06[M-H-C14H18O9]-,161.05[M-H-C17H18O7]-137.02[M-H-C16H22O9] |
| 27 | Chlorogenic acid isomer | 9.93 | C_16_H_18_O_9_ | N | 353.08780 | 353.08737 | -1.233 | 191.06[M-H-C9H6O3]-,179.03[M-H-C7H10O5]-,135.04[M-H-C7H10O5-CO2]-,109.03[M-H-C7H10O5-CO2-C2H2]-, |
| 28 | Chlorogenic acid^*^ | 9.93 | C_16_H_18_O_9_ | N | 353.08780 | 353.08737 | -1.233 | 191.06[M-H-C9H6O3]-,179.03[M-H-C7H10O5]-,135.04[M-H-C7H10O5-CO2]-,109.03[M-H-C7H10O5-CO2-C2H2]-, |
| 29 | Chlorogenic acid isomer | 9.93 | C_16_H_18_O_9_ | N | 353.08780 | 353.08737 | -1.233 | 191.06[M-H-C9H6O3]-,179.03[M-H-C7H10O5]-,135.04[M-H-C7H10O5-CO2]- |
| 30 | Caffeic acid^*^ | 10.77 | C_9_H_8_O_4_ | N | 179.03498 | 179.03473 | -1.408 | 135.04[M-H-CO2]- |
| 31 | 6’-O-galloyl-Desbenzoylpaeoniflorin Isomer | 11.02 | C_23_H_28_O_14_ | N | 527.14062 | 527.13995 | -1.287 | 491.12[M-H-H2O-H2O]-,345.12[M-H-C8H6O5]-,313.06[M-H-C10H14O5]-,271.05[M-H-C10H14O5-C2H2O]-,211.03[M-H-C10H14O5-C2H2O-C2H4O2]-,169.01[M-H-C10H14O5-C6H8O4],125.02[M-H-C10H14O5-C6H8O4-CO2]- |
| 32 | Carthamidin/isocarthamidin-2glu/gal | 12.12 | C_27_H_32_O_16_ | P | 613.17631 | 613.17548 | -1.356 | 451.12[M+H-C6H10O5]+,331.08[M+H-C6H10O5-C4H8O4]+,289.07[M+H-C6H10O5-C6H10O5]+,211.02[M+H-C6H10O5-C6H10O5-C6H6]+,147.04[M+H-C6H10O5-C6H10O5-C6H6O4]+, |
| 33 | Hydroxysafflor yellow A^*^ | 12.20 | C_27_H_32_O_16_ | N | 611.16175 | 611.16083 | -1.518 | 491.12[M-H-C4H8O4]-,473.11[M-H-C4H8O4-H2O]-,403.10[M-H-C6H10O5-CH2O2]-,325.07[M-H-C6H10O5-CH2O2-C2H4O2-H2O]-,295.06[M-H-C6H10O5-CH2O2-C2H4O2-H2O-CH2O]-,119.05[M-H-C19H24O15]- |
| 34 | Senkyunolide J/N isomer | 12.94 | C_12_H_18_O_4_ | P | 227.12778 | 227.12741 | -1.654 | 209.12[M+H-H2O]+,191.11[M+H-H2O-H2O]+,167.11[M+H-CH2O2-CH2]+,149.10[M+H-CH2O2-CH2-H2O]+,131.09[M+H-CH2O2-CH2-H2O-H2O]+, |
| 35 | Carthamidin/isocarthamidin-glu/gal isomer | 13.86 | C_21_H_22_O_11_ | P | 451.12348 | 451.12289 | -1.325 | 289.07[M+H-C6H10O5]+,169.01[M+H-C6H10O5-C8H8O]+,147.02[M+H-C6H10O5-C6H6O4]+,119.05[M+H-C6H10O5-C7H6O5]+ |
| 36 | Quercetin-O-2glu/gal isomer | 14.13 | C_27_H_30_O_17_ | P | 627.15557 | 627.15472 | -1.364 | 465.10[M+H-C6H10O5]+,303.05[M+H-C6H10O5-C6H10O5]+,127.04[M+H-C6H10O5-C6H10O5-C9H4O4]+,109.03[M+H-C6H10O5-C6H10O5-C9H6O5]+,85.03[M+H-C6H10O5-C6H10O5-C9H6O5-C2]+,69.03[M+H-C6H10O5-C6H10O5-C9H6O5-C-CO] |
| 37 | Quercetin-O-2glu/gal isomer | 14.13 | C_27_H_30_O_17_ | P | 627.15557 | 627.15472 | -1.364 | 465.10[M+H-C6H10O5]+,303.05[M+H-C6H10O5-C6H10O5]+,127.04[M+H-C6H10O5-C6H10O5-C9H4O4]+,109.03[M+H-C6H10O5-C6H10O5-C9H6O5]+,85.03[M+H-C6H10O5-C6H10O5-C9H6O5-C2]+, |
| 38 | Quercetin-O-2glu/gal isomer | 14.13 | C_27_H_30_O_17_ | P | 627.15557 | 627.15472 | -1.364 | 465.10[M+H-C6H10O5]+,303.05[M+H-C6H10O5-C6H10O5]+,127.04[M+H-C6H10O5-C6H10O5-C9H4O4]+,85.03[M+H-C6H10O5-C6H10O5-C9H6O5-C2]+ |
| 39 | Quercetin-O-2glu/gal isomer | 14.13 | C_27_H_30_O_17_ | P | 627.15557 | 627.15472 | -1.364 | 465.10[M+H-C6H10O5]+,303.05[M+H-C6H10O5-C6H10O5]+,127.04[M+H-C6H10O5-C6H10O5-C9H4O4]+,85.03[M+H-C6H10O5-C6H10O5-C9H6O5-C2]+ |
| 40 | Quercetin-O-2glu/gal isomer | 14.13 | C_27_H_30_O_17_ | P | 627.15557 | 627.15472 | -1.364 | 465.10[M+H-C6H10O5]+,303.05[M+H-C6H10O5-C6H10O5]+,145.03[M+H-C6H10O5-C6H10O5-C9H2O3]+,127.04[M+H-C6H10O5-C6H10O5-C9H4O4]+,109.03[M+H-C6H10O5-C6H10O5-C9H6O5]+,85.03[M+H-C6H10O5-C6H10O5-C9H6O5-C2]+, |
| 41 | Kaempferol-O-glu/gal+glu A | 14.54 | C_27_H_30_O_16_ | P | 611.16066 | 611.15887 | -2.931 | 449.11[M+H-C6H10O5]+,287.05[M+H-C6H10O5-C6H10O5]+,169.01[M+H-C6H10O5-C6H10O5-C8H6O]+,123.01[M+H-C6H10O5-C6H10O5-C9H8O3]+ |
| 42 | Albiflorin | 14.61 | C_23_H_28_O_11_ | N | 479.15588 | 479.15494 | -1.972 | 327.11[M-H-CH2O-C7H6O2]-,121.03[M-H-C16H22O9]- |
| 43 | Paeoniflorin^*^ | 14.61 | C_23_H_28_O_11_ | N | 479.15588 | 479.15494 | -1.972 | 449.14[M-H-CH2O]-,367.12,357.18[M-H-C7H6O2]-,161.05[M-H-C17H18O6]-,121.03[M-H-C16H22O9]-, |
| 44 | Paeoniflorin Isomer | 14.61 | C_23_H_28_O_11_ | N | 479.15588 | 479.15494 | -1.972 | 327.11[M-H-C8H8O3]-,165.06[M-H-C8H8O3-C6H10O5]-,161.05[M-H-C8H8O3-C9H10O3]-121.03[M-H-C16H22O9]- |
| 45 | Paeoniflorin Isomer | 14.61 | C_23_H_28_O_11_ | N | 479.15588 | 479.15494 | -1.972 | 327.11[M-H-C8H8O3]-,263.07,177.06[M-H-C7H6O2-C6H10O5]-,165.06[M-H-C8H8O3-C6H10O5]-,121.03[M-H-C16H22O9]-, |
| 46 | Ethyl gallate isomer | 14.70 | C_9_H_10_O_5_ | N | 197.04554 | 197.04501 | -2.724 | 169.01[M-H-C2H4]-,125.02[M-H-C2H4-CO2]- |
| 47 | Salvianic acid A isomer | 14.70 | C_9_H_10_O_5_ | N | 197.04554 | 197.04501 | -2.724 | 169.01[M-H-C2H4]-,140.01[M-H-C2H4-CHO]-,125.02[M-H-C2H4-CO2]-,111.01[M-H-C2H4-CO2-CH2]- |
| 48 | Coniferyl aldehyde | 15.35 | C_10_H_10_O_3_ | N | 177.05571 | 177.05528 | -2.471 | 162.03[M-H-CH3]-,149.06[M-H-CO]-,135.05[M-H-CO-CH2]-,107.05[M-H-CO-CH2-CO]- |
| 49 | Quercetin-isomer | 15.59 | C_15_H_10_O_7_ | P | 303.04992 | 303.04926 | -2.208 | 285.04[M+H-H2O]+,275.02,257.02[M+H-CH2O2]+ |
| 50 | Quercetin-isomer | 15.59 | C_15_H_10_O_7_ | P | 303.04992 | 303.04926 | -2.208 | 257.04[M+H-CH2O2]+,229.05[M+H-CH2O2-CO]+,165.02,153.02,137.02,109.03， |
| 51 | Quercetin isomer | 15.59 | C_15_H_10_O_7_ | P | 303.04992 | 303.04926 | -2.208 | 257.04[M+H-CH2O2]+,165.01776,153.01775, 137.02286, 105.03358 |
| 52 | Hyperin | 15.59 | C_21_H_20_O_12_ | P | 465.10275 | 465.10233 | -0.908 | 303.05[M+H-C6H10O5]+,181.01[M+H-C6H10O5-C7H6O2]+,153.02[M+H-C6H10O5-C8H6O3]+,123.01[M+H-C6H10O5-C9H8O4]+,121.03[M+H-C6H10O5-C8H6O5]+,109.03[M+H-C6H10O5-C9H6O5]+ |
| 53 | Quercetin | 15.59 | C_15_H_10_O_7_ | P | 303.04992 | 303.04926 | -2.208 | 181.01[M+H-C7H6O2]+,123.01[M+H-C9H8O4]+,121.03[M+H-C8H6O5]+ |
| 54 | Rutin isomer | 15.74 | C_27_H_30_O_16_ | P | 611.16066 | 611.15985 | -1.327 | 449.11,287.05[M+H-C6H10O5-C6H10O5]+,145.05[M+H-C6H10O5-C6H10O5-C9H2O2]+ |
| 55 | Rutin isomer | 15.74 | C_27_H_30_O_16_ | P | 611.16066 | 611.15985 | -1.327 | 449.11,287.05[M+H-C6H10O5-C6H10O5]+,163.06, 153.02[M+H-C6H10O5-C6H10O5-C8H6O2]+, 145.05[M+H-C6H10O5-C6H10O5-C9H2O2]+, 109.03[M+H-C6H10O5-C6H10O5-C9H6O4]+, 85.03[M+H-C6H10O5-C6H10O5-C9H6O4-C2]+ |
| 56 | Rutin^*^ | 15.74 | C_27_H_30_O_16_ | P | 611.16066 | 611.15985 | -1.327 | 465.09589, 303.05[M+H-C6H10O4-C6H10O5]+, 153.02[M+H-C6H10O4-C6H10O5-C8H6O3]+, 145.05[M+H-C6H10O4-C6H10O5-C9H2O3]+, 109.03[M+H-C6H10O4-C6H10O5-C9H6O5]+, |
| 57 | Ferulic Acid^*^ | 15.85 | C_10_H_10_O_4_ | P | 195.06518 | 195.06502 | -0.848 | 177.05[M+H-H2O]+,163.04[M+H-H2O-CH2]+,145.03[M+H-H2O-CH4O]+,135.04[M+H-H2O-CH2-CO]+,117.03[M+H-H2O-CH2-CO-H2O]+ |
| 58 | Carthamidin/isocarthamidin-glu/gal isomer | 16.07 | C_21_H_22_O_11_ | P | 451.12348 | 451.12265 | -1.857 | 289.07[M+H-C6H10O5]+, 169.01[M+H-C6H10O5-C8H8O]+, 147.02[M+H-C6H10O5-C6H6O4]+, 119.05[M+H-C6H10O5-C7H6O5]+ |
| 59 | Hyperin isomer | 16.87 | C_21_H_20_O_12_ | P | 465.10275 | 465.10178 | -2.091 | 303.05[M+H-C6H10O5]+,181.01[M+H-C6H10O5-C7H6O2]+,153.02[M+H-C6H10O5-C8H6O3]+, 123.01[M+H-C6H10O5-C9H8O4]+,121.03[M+H-C6H10O5-C8H6O5]+,109.03[M+H-C6H10O5-C9H6O5]+ |
| 60 | Galloylpaeoniflorin isomer | 17.51 | C_30_H_32_O_15_ | N | 631.16684 | 631.16608 | -1.209 | 613.16[M-H-H2O],491.12[M-H-H2O-C7H6O2]-,399.09[M-H-H2O-C7H6O2-C6H4O]-,313.06[M-H-H2O-C7H6O2-C10H10O3]-,271.05[M-H-H2O-C7H6O2-C10H10O3-C2H2O]-,211.02[M-H-H2O-C7H6O2-C10H10O3-C2H2O-C2H4O2]]-,169.01[M-H-C23H26O10]-,121.03[M-H-H2O-C23H24O12]- |
| 61 | Galloylpaeoniflorin isomer | 17.51 | C_30_H_32_O_15_ | N | 631.16684 | 631.16608 | -1.209 | 613.16[M-H-H2O]-,491.12[M-H-H2O-C7H6O2]-,399.09[M-H-H2O-C7H6O2-C6H4O]-,313.06[M-H-H2O-C7H6O2-C10H10O3]-,271.05[M-H-H2O-C7H6O2-C10H10O3-C2H2O]-,211.02[M-H-H2O-C7H6O2-C10H10O3-C2H2O-C2H4O2]]-,169.01[M-H-C23H26O10]-,121.03[M-H-H2O-C23H24O12]- |
| 62 | Galloylpaeoniflorin isomer | 17.51 | C_30_H_32_O_15_ | N | 631.16684 | 631.16684 | -1.209 | 613.16[M-H-H2O]-,491.12[M-H-H2O-C7H6O2]-,399.09[M-H-H2O-C7H6O2-C6H4O]-,313.06[M-H-H2O-C7H6O2-C10H10O3]-,271.05[M-H-H2O-C7H6O2-C10H10O3-C2H2O]-,211.02[M-H-H2O-C7H6O2-C10H10O3-C2H2O-C2H4O2]]-,169.01[M-H-C23H26O10]-,121.03[M-H-H2O-C23H24O12]- |
| 63 | Galloylpaeoniflorin isomer | 17.51 | C_30_H_32_O_15_ | N | 631.16684 | 631.16608 | -1.209 | 431.13,313.06[M-H-H2O-C7H6O2-C10H10O3]-,169.01[M-H-C23H26O10]-,121.03[M-H-H2O-C23H24O12]- |
| 64 | Carthamidin/isocarthamidin-glu/gal isomer | 17.62 | C_21_H_22_O_11_ | P | 451.12348 | 451.12238 | -2.456 | 289.07[M+H-C6H10O5]+,169.01[M+H-C6H10O5-C8H8O]+,147.02[M+H-C6H10O5-C6H6O4]+,119.05[M+H-C6H10O5-C7H6O5]+ |
| 65 | Vanillin | 17.71 | C_8_H_8_O_3_ | N | 151.04006 | 151.03972 | -2.300 | 135.01[M-H-CH4]-,109.03[M-H-C2H2O]-,91.02[M-H-C2H2O-H2O]- |
| 66 | p-Anisic acid | 17.71 | C_8_H_8_O_3_ | N | 151.04006 | 151.03972 | -2.300 | 135.01[M-H-CH4]-,109.03[M-H-C2H2O]- |
| 67 | Luteolin/kaempferol isomer | 17.96 | C_15_H_10_O_6_ | P | 287.05501 | 287.05444 | -2.001 | 269.04, 247.10, 165.02[M+H-C7H6O2]+, 121.03[M+H-C7H6O2-CO2]+ |
| 68 | Luteolin | 17.96 | C_15_H_10_O_6_ | P | 287.05501 | 287.05444 | -2.001 | 153.02[M+H-C8H6O2]+, 135.04 |
| 69 | Kaempferol^*^ | 17.96 | C_15_H_10_O_6_ | P | 287.05501 | 287.05444 | -2.001 | 258.05[M+H-CHO]+,153.02[M+H-C8H6O2]+,133.03[M+H-C7H6O4]+ |
| 70 | Perlolyrine | 17.97 | C_16_H_12_N_2_O_2_ | P | 265.09715 | 265.09680 | -1.336 | 247.09[M+H-H2O]+,219.09[M+H-H2O-CO]+,206.08[M+H-H2O-CO-CH]+,185.07[M+H-H2O-C5H2]+, |
| 71 | Kaempferol-rut | 17.97 | C_27_H_30_O_15_ | P | 595.16574 | 595.16504 | -1.187 | 449.10[M+H-C6H10O5]+,287.05[M+H-C6H10O5-C6H10O4]+,153.02[M+H-C6H10O5-C6H10O4-C8H6O2]+,137.02[M+H-C6H10O5-C6H10O4-C8H6O3]+,121.03[M+H-C6H10O5-C6H10O4-C8H6O4]+,109.03[M+H-C6H10O5-C6H10O4-C8H6O2-CO2]+ |
| 72 | Kaempferol-O-Glc-isomer | 18.51 | C_21_H_20_O_11_ | P | 449.10783 | 449.10706 | -1.732 | 287.05[M+H-C6H10O5]+,153.02[M+H-C6H10O5-C8H6O2]+,121.03[M+H-C6H10O5-C8H6O4]+,109.03[M+H-C6H10O5-C8H6O2-CO2]+ |
| 73 | Luteolin-glc isomer | 18.51 | C_21_H_20_O_11_ | P | 449.10783 | 449.10706 | -1.732 | 287.05[M+H-C6H10O5]+ |
| 74 | Luteolin-O-glc | 18.51 | C_21_H_20_O_11_ | P | 449.10783 | 449.10706 | -1.732 | 391.21,287.05[M+H-C6H10O5]+,153.02[M+H-C6H10O5-C8H6O2]+, |
| 75 | Kaempferol-O-Glc isomer | 18.51 | C_21_H_20_O_11_ | P | 449.10783 | 449.10706 | -1.732 | 287.05[M+H-C6H10O5]+,153.02[M+H-C6H10O5-C8H6O2]+,109.03[M+H-C6H10O5-C8H6O2-CO2]+ |
| 76 | Kaempferol-O-Glc isomer | 18.51 | C_21_H_20_O_11_ | P | 449.10783 | 449.10706 | -1.732 | 287.05[M+H-C6H10O5]+,153.02[M+H-C6H10O5-C8H6O2]+,121.03[M+H-C6H10O5-C8H6O4]+,109.03[M+H-C6H10O5-C8H6O2-CO2]+ |
| 77 | Kaempferol-O-Glc isomer | 18.51 | C_21_H_20_O_11_ | P | 449.10783 | 449.10706 | -1.732 | 287.05[M+H-C6H10O5]+,153.02[M+H-C6H10O5-C8H6O2]+,109.03[M+H-C6H10O5-C8H6O2-CO2]+ |
| 78 | SenkyunolideG/K | 18.53 | C_12_H_16_O_3_ | P | 209.11722 | 209.11700 | -1.056 | 191.11[M+H-H2O],173.10[M+H-H2O-H2O]+,163.11[M+H-H2O-CO]+,149.06[M+H-H2O-C3H6]+,145.10[M+H-H2O-CO-H2O]+,135.04[M+H-H2O-CO-CO]+, |
| 79 | Senkyunolide J/N isomer | 18.55 | C_12_H_18_O_4_ | P | 227.12778 | 227.12744 | -1.521 | 209.12[M+H-H2O]+,191.11[M+H-H2O-H2O]+163.11[M+H-H2O-H2O-CO]+,153.05[M+H-H2O-C4H8]+, |
| 80 | Senkyunolide J/N isomer | 18.55 | C_12_H_18_O_4_ | P | 227.12778 | 227.12744 | -1.521 | 209.12[M+H-H2O]+,191.11[M+H-H2O-H2O]+163.11[M+H-H2O-H2O-CO]+,153.05[M+H-H2O-C4H8]+, |
| 81 | Senkyunolide J/N isomer | 18.55 | C_12_H_18_O_4_ | P | 227.12778 | 227.12744 | -1.521 | 209.12[M+H-H2O]+,191.11[M+H-H2O-H2O]+,163.11[M+H-H2O-H2O-CO]+,153.05[M+H-H2O-C4H8]+, |
| 82 | Azelaic acid | 19.62 | C_9_H_16_O_4_ | N | 187.09758 | 187.09726 | -1.722 | 169.09[M-H-H2O]-,143.11[M-H-CO2]-,125.10[M-H-CO2-H2O]- |
| 83 | Carthamidin/isocarthamidin-glu/gal isomer | 20.09 | C_21_H_22_O_11_ | P | 451.12348 | 451.12253 | -2.123 | 289.07[M+H-C6H10O5]+,169.01[M+H-C6H10O5-C8H8O]+,147.02[M+H-C6H10O5-C6H6O4]+,119.05[M+H-C6H10O5-C7H6O5]+ |
| 84 | Rosmarinic acid^*^ | 20.18 | C_18_H_16_O_8_ | N | 359.07724 | 359.07672 | -1.45 | 197.05[M-H-C9H6O3]-,179.03[M-H-C9H6O3-H2O]-,161.02[M-H-C9H10O5]-,135.04[M-H-C9H6O3-H2O-CO2]- |
| 85 | Salvianolic acid B^*^ | 20.22 | C_36_H_30_O_16_ | N | 717.14610 | 717.14453 | -2.200 | 359.08[M-H-C9H8O4-C9H6O4]-,313.07[M-H-C9H8O4-C9H6O4-CH2O2]-,197.05[M-H-C27H20O11]-,179.03[M-H-C27H20O11-H2O]-,161.02[M-H-C27H20O11-H2O-H2O]-,151.04[M-H-C27H20O11-H2O-CO]-,133.03[M-H-C27H20O11-H2O-H2O-CO]-,123.05[M-H-C27H20O11-H2O-CO-CO]- |
| 86 | Senkyunolide I/H isomer | 21.03 | C_12_H_16_O_4_ | P | 225.11213 | 225.11172 | -1.846 | 207.10[M+H-H2O]+,165.09[M+H-CH2O2-CH2]+,151.04[M+H-H2O-CO-CO]+,137.10[M+H-H2O-CO-CO-CH2]+,123.04[M+H-H2O-CO-C4H8]+,107.05[M+H-H2O-CO-C4H8O]+,95.05[M+H-H2O-CO-C4H8-CO]+ |
| 87 | Senkyunolide I/H isomer | 21.03 | C_12_H_16_O_4_ | P | 225.11213 | 225.11172 | -1.846 | 207.10[M+H-H2O]+,165.09[M+H-CH2O2-CH2]+,151.04[M+H-H2O-CO-CO]+,137.10[M+H-H2O-CO-CO-CH2]+,123.04[M+H-H2O-CO-C4H8]+,107.05[M+H-H2O-CO-C4H8O]+,95.05[M+H-H2O-CO-C4H8-CO]+ |
| 88 | Senkyunolide F isomer | 21.03 | C_12_H_14_O_3_ | P | 207.10157 | 207.10130 | -1.308 | 189.09[M+H-H2O]+,161.10[M+H-H2O-CO]+,147.08[M+H-H2O-CO-CH2]+,119.09[M+H-H2O-CO-CH2-CO]+, |
| 89 | Senkyunolide I/H isomer | 21.03 | C_12_H_16_O_4_ | P | 225.11213 | 225.11172 | -1.846 | 207.10[M+H-H2O]+,189.09[M+H-H2O-H2O]+,165.09[M+H-CH2O2-CH2]+,161.10[M+H-H2O-H2O-CO]+,147.08[M+H-H2O-H2O-CO-CH2]+,133.10[M+H-H2O-H2O-CO-CO]+,119,.09[M+H-H2O-H2O-CO-CO-CH2]+, |
| 90 | Senkyunolide I^*^ | 21.03 | C_12_H_16_O_4_ | P | 225.11213 | 225.11172 | -1.846 | 207.10[M+H-H2O]+,189.09[M+H-H2O-H2O]+,165.09[M+H-CH2O2-CH2]+,161.10[M+H-H2O-H2O-CO]+,147.08[M+H-H2O-H2O-CO-CH2]+,133.10[M+H-H2O-H2O-CO-CO]+,119,.09[M+H-H2O-H2O-CO-CO-CH2]+, |
| 91 | Senkyunolide F isomer | 21.03 | C_12_H_14_O_3_ | P | 207.10157 | 207.10130 | -1.308 | 189.09[M+H-H2O]+,161.10[M+H-H2O-CO]+,147.08[M+H-H2O-CO-CH2]+,133.10[M+H-H2O-CO-CO]+, |
| 92 | Senkyunolide F isomer | 21.03 | C_12_H_14_O_3_ | P | 207.10157 | 207.10130 | -1.308 | 189.09[M+H-H2O]+,161.10[M+H-H2O-CO]+,147.08[M+H-H2O-CO-CH2]+,133.10[M+H-H2O-CO-CO]+, |
| 93 | Benzoyloxypaeoniflorin Isomer | 21.87 | C_30_H_32_O_13_ | N | 599.17701 | 599.17627 | -1.242 | 477.14[M-H-C7H6O2]-,431.13[M-H-C7H6O2-CH2O2]-,281.07[M-H-C7H6O2-C10H12O4]-,239.05[M-H-C7H6O2-C10H12O4-C2H2O]-,137.02[M-H-C23H26O10]-,121.03[M-H-C23H26O11]- |
| 94 | Benzoyloxypaeoniflorin | 21.87 | C_30_H_32_O_13_ | N | 599.17701 | 599.17627 | -1.242 | 477.14[M-H-C7H6O2]-,385.09,333.10,281.07[M-H-C7H6O2-C10H12O4]-,165.06[M-H-C7H6O2-C10H12O4-C4H4O4]-,137.02[M-H-C23H26O10]-,121.03[M-H-C23H26O11]- |
| 95 | 3,7- or 3,8-Dimethyl ellagic acid isomer | 23.29 | C_16_H_10_O_8_ | N | 329.03029 | 329.02975 | -1.643 | 314.01[M-H-CH3]-,298.98[M-H-CH3-CH3]-.270.99[M-H-CH3-CH3-CO]-, |
| 96 | 3,7- or 3,8-Dimethyl ellagic acid isomer | 23.29 | C_16_H_10_O_8_ | N | 329.03029 | 329.02975 | -1.643 | 314.01[M-H-CH3]-,298.98[M-H-CH3-CH3]-.270.99[M-H-CH3-CH3-CO]- |
| 97 | Ethyl4-hydroxy-3-methoxycinnamate | 24.23 | C_12_H_14_O_4_ | N | 221.08193 | 221.08156 | -1.683 | 177.09[M-H-CO2]-,135.04[M-H-CO2-C3H6]-,108.02[M-H-CO2-C4H6-CH3]- |
| 98 | Salvianolic acid A isomer | 24.24 | C_26_H_22_O_10_ | N | 493.11402 | 493.11267 | -2.738 | 295.06[M-H-C9H10O5]-,197.04[M-H-C17H12O5]-,185.02[M-H-C9H10O5-C6H6O2]-,179.04[M-H-C17H12O5-H2O]-,135.04[M-H-C17H12O5-H2O-CO2]-,109.03[M-H-C17H12O5-H2O-CO2-C2H2]-, |
| 99 | Salvianolic acid A^*^ | 24.24 | C_26_H_22_O_10_ | N | 493.11402 | 493.11267 | -2.738 | 295.06[M-H-C9H10O5]-,197.04[M-H-C17H12O5]-,185.02[M-H-C9H10O5-C6H6O2]-,179.04[M-H-C17H12O5-H2O]-,135.04[M-H-C17H12O5-H2O-CO2]-,109.03[M-H-C17H12O5-H2O-CO2-C2H2]-, |
| 100 | SenkyunolideB/C/E isomer | 24.27 | C_12_H_12_O_3_ | P | 205.08592 | 205.08563 | -1.418 | 187.08[M+H-H2O]+,177.09[M+H-CO]+,163.04[M+H-C3H6]+,149.02[M+H-C4H8]+, |
| 101 | SenkyunolideB/C/E isomer | 24.27 | C_12_H_12_O_3_ | P | 205.08592 | 205.08563 | -1.418 | 187.08[M+H-H2O]+,177.09[M+H-CO]+,163.04[M+H-C3H6]+,149.02[M+H-C3H6-CH2]+, |
| 102 | SenkyunolideB/C/E isomer | 24.27 | C_12_H_12_O_3_ | P | 205.08592 | 205.08563 | -1.418 | 187.08[M+H-H2O]+,169.06[M+H-H2O-H2O]+,159.08[M+H-H2O-CO]+,149.02[M+H-C4H8]+, |
| 103 | Salvianolic acid C isomer | 25.03 | C_26_H_20_O_10_ | N | 491.09837 | 491.09796 | -0.835 | 311.06[M-H-C9H8O4]-,293.05[M-H-C9H10O5]-,197.05[M-H-C17H10O5]-,179.03[M-H-C17H10O5-H2O]-,135.05[M-H-C17H10O5-H2O-CO2]-,109.03[M-H-C17H10O5-H2O-CO2-C2H2]- |
| 104 | Salvianolic acid C isomer | 25.03 | C_26_H_20_O_10_ | N | 491.09837 | 491.09796 | -0.835 | 311.06[M-H-C9H8O4]-,293.05[M-H-C9H10O5]-,197.05[M-H-C17H10O5]-,179.03[M-H-C17H10O5-H2O]-,135.05[M-H-C17H10O5-H2O-CO2]- |
| 105 | Naringenin^*^ | 25.96 | C_15_H_12_O_5_ | P | 273.07575 | 273.07510 | -2.380 | 153.02[M+H-C8H8O]+,147.04[M+H-C6H6O3]+,119.05[M+H-C7H6O4]+ |
| 106 | Apigenin | 26.13 | C_15_H_10_O_5_ | P | 271.06009 | 271.05945 | -2.398 | 153.02[M+H-C8H6O]+,119.05[M+H-C7H4O4]+, |
| 107 | Benzoylpaeoniflorin^*^ | 26.47 | C_30_H_32_O_12_ | N | 583.18209 | 583.17999 | -3.617 | 165.05[M-H-C21H22O9]-,135.05[M-H-C21H22O9-CH2O]-,121.03[M-H-C23H26O10]- |
| 108 | E/Z-Butylidenephthalide | 29.76 | C_12_H_12_O_2_ | P | 189.09100 | 189.09076 | -1.302 | 171.08[M+H-H2O]+,161.10[M+H-CO]+,153.07[M+H-H2O-H2O]+,143.09[M+H-CO-H2O]+, |
| 109 | E/Z-Butylidenephthalide | 29.76 | C_12_H_12_O_2_ | P | 189.09100 | 189.09076 | -1.302 | 171.08[M+H-H2O]+,153.07[M+H-H2O-H2O]+,147.04[M+H-C3H6],143.09[M+H-H2O-CO]+,133.03[M+H-C4H8]+,117.07[M+H-H2O-CO-C2H2]+,77.04[[M+H-H2O-CO-C2H2-C3H4]+ |
| 110 | Butylidenephthalide isomer | 29.76 | C_12_H_12_O_2_ | P | 189.09100 | 189.09076 | -1.302 | 171.08[M+H-H2O]+,161.10[M+H-CO]+,143.09[M+H-CO-H2O]+,133.03[M+H-C4H8]+, |
| 111 | Butylidenephthalide | 29.76 | C_12_H_12_O_2_ | P | 189.09100 | 189.09076 | -1.302 | 171.08[M+H-H2O]+,161.10[M+H-CO],153.07[M+H-H2O-H2O]+,149.02,133.03[M+H-C4H8]+ |
| 112 | Neocnidilide | 29.89 | C_12_H_18_O_2_ | P | 195.13795 | 195.13770 | -1.314 | 177.13[M+H-H2O]+,167.14,159.12[M+H-H2O-H2O]+,81.07[M+H-H2O-H2O-C6H6]+, |
| 113 | Ligustilides isomer | 30.04 | C_12_H_14_O_2_ | P | 191.10665 | 191.10635 | -1.603 | 149.06[M+H-C3H6]+,135.04[M+H-C4H8]+ |
| 114 | Ligustilide | 30.04 | C_12_H_14_O_2_ | P | 191.10665 | 191.10635 | -1.603 | 173.10[M+H-H2O]+,163.11[M+H-CO]+,145.10[M+H-CO-H2O]+,135.04[M+H-C4H8]+, |
| 115 | Ethyl ferulate | 30.78 | C_12_H_14_O_4_ | N | 221.08193 | 221.08145 | -2.181 | 177.09[M-H-CO2]-,149.10[M-H-CO2-CO]-,134.04[M-H-CO2-CO-CH3]-,121.03[M-H-CO2-CO-CH3-CH]-,71.05[M-H-C8H6O3]-,69.03[M-H-C8H8O3]- |
| 116 | Senkyunolide A | 35.15 | C_12_H_16_O_2_ | P | 193.12230 | 193.12216 | -0.758 | 175.11[M+H-H2O]+,147.12[[M+H-H2O-CO]+,137.06[M+H-C4H8]+,93.07[M+H-C4H8-CO2]+, |
| 117 | Senkyunolide A isomer | 35.15 | C_12_H_16_O_2_ | P | 193.12230 | 193.12216 | -0.758 | 175.11[M+H-H2O]+,147.12[M+H-H2O-CO]+,137.06[M+H-C4H8]+, |
| 118 | Senkyunolide A isomer | 35.29 | C_12_H_16_O_2_ | P | 193.12230 | 193.12227 | -0.188 | 175.11[M+H-H2O]+,165.13[M+H-CO]+,149.02,137.06[M+H-C4H8]+,85.07,57.07 |
| 119 | Senkyunolide A isomer | 35.29 | C_12_H_16_O_2_ | P | 193.12230 | 193.12227 | -0.188 | 175.11[M+H-H2O]+,165.13[M+H-CO]+147.12[M+H-CO-H2O]+,137.06[M+H-C4H8]+,85.07,57.07 |
| 120 | Miltiodiol | 36.55 | C_19_H_22_O_3_ | P | 299.16417 | 299.16360 | -1.909 | 281.15,257.12[M+H-C3H6]+,255.17[M+H-CO2]+, |
| 121 | Deoxyneocryptotanshinone | 36.55 | C_19_H_22_O_3_ | P | 299.16417 | 299.16360 | -1.909 | 281.15,257.12[M+H-C3H6]+,255.17[M+H-CO2]+,253.16[M+H-CO-H2O]+,239.11[M+H-C3H6-H2O]+,229.09 |
| 122 | Cryptotanshinone isomer | 37.10 | C_19_H_20_O_3_ | P | 297.14852 | 297.14804 | -1.619 | 269.15[M+H-CO]+,253.16[M+H-CO2]+,238.13[M+H-CO2-CH3]+,225.16[M+H-CO2-CO]+,211.11[M+H-CO2-C3H6]+ |
| 123 | Tanshinone V | 37.10 | C_19_H_22_O_4_ | P | 315.15908 | 315.15839 | -2.207 | 297.15[M+H-H2O]+,287.16,269.15[M+H-H2O-CO]+,251.14[[M+H-H2O-CO-H2O]+,213.09[M+H-H2O-CO-C4H8]+ |
| 124 | Tanshinone V-isomer | 37.10 | C_19_H_22_O_4_ | P | 315.15908 | 315.15839 | -2.207 | 297.15[M+H-H2O]+,279.23,253.16[M+H-H2O-CO2]+,238.13[M+H-H2O-CO2-CH3]+,225.16[M+H-H2O-CO2-CO]+, |
| 125 | Cryptotanshinone | 37.10 | C_19_H_20_O_3_ | P | 297.14852 | 297.14804 | -1.619 | 279.14,269.15[M+H-CO]+,253.16[M+H-CO2]+,251.14[M+H-CH2O2]+,238.13[M+H-CO2-CH3]+,225.16[M+H-CO2-CO]+,211.11[M+H-CO2-C3H6]+, |
| 126 | Dihydrotanshinone I | 37.78 | C_18_H_14_O_3_ | P | 279.10157 | 279.10120 | -1.329 | 261.09[M+H-H2O]+,233.10[M+H-H2O-CO]+,149.02[M+H-C10H10]+ |
| 127 | Tetramethylpyrazine | 56.61 | C_8_H_12_N_2_ | P | 137.10732 | 137.10728 | -0.328 | 122.08[M+H-CH3]+ |

^*^ Structures confirmed by comparison with reference standards.
