# Supplementary material for: Metabolomic Insights Into the Synergistic Effect of Biapenem in Combination With Xuebijing Injection Against Sepsis
Source: Front Pharmacol. 2020 Apr 22;11:502. doi: 10.3389/fphar.2020.00502 (PMC7189733; doi:10.3389/fphar.2020.00502)
Supplement: Supplementary file 4 [file Table_3.docx]

**Supplementary Table S3** Regression equation, linear range, LOQs and LODs of the developed method.

| **Compound** | **Regression equation** | **Correlation coefficient  (R^2^)** | **Linear range  (μg/mL)** | **LOQ  (ng/mL)** | **LOD  (ng/mL)** |
| --- | --- | --- | --- | --- | --- |
| Hydroxysafflor yellow A | y = 4.22×106 x + 1.42×107 | 0.9991 | 20.684-661.879 | 15.78 | 4.73 |
| Oxypaeoniflorin | y = 3.26×107 x + 7.64×106 | 0.9990 | 0.654-20.940 | 10.33 | 3.10 |
| Benzoylpaeoniflorin | y = 6.51×107 x + 4.85×105 | 0.9998 | 0.266-8.508 | 2.14 | 0.64 |
| Senkyunolide I | y = 2.55×107 x + 1.38×106 | 0.9992 | 0.274-8.773 | 3.53 | 1.06 |
| Succinic acid | y = 5.26×10^7^ x + 6.18×10^6^ | 0.9997 | 0.182-5.840 | 1.89 | 0.57 |
| Gallic acid | y = 1.57×108 x + 1.10×107 | 0.9991 | 0.161-5.164 | 0.22 | 0.07 |
| Rosmarinic acid | y = 4.25×108 x - 9.20×106 | 0.9997 | 0.056-1.790 | 0.14 | 0.04 |
| Caffeic acid | y = 1.93×108 x + 3.15×106 | 0.9993 | 0.046-1.470 | 0.44 | 0.13 |
| Protocatechuic aldehyde | y = 1.22×108 x + 1.19×106 | 0.9996 | 0.057-1.828 | 1.54 | 0.46 |
| Protocatechuic acid | y = 3.35×107 x + 1.30×106 | 0.9996 | 0.078-2.502 | 4.14 | 1.24 |
| Rutin | y = 1.51×108 x - 5.46×106 | 0.9990 | 0.046-1.474 | 1.22 | 0.37 |
| Salvianic acid A | y = 1.86×108 x - 1.24×106 | 0.9999 | 0.054-1.720 | 0.22 | 0.06 |
| Chlorogenic acid | y = 2.10×108 x + 1.26×106 | 0.9990 | 0.048-1.542 | 0.19 | 0.06 |
| Naringenin | y = 6.73×108 x - 1.16×106 | 0.9995 | 0.005-0.148 | 0.36 | 0.11 |
