# Supplementary material for: Metabolomic Insights Into the Synergistic Effect of Biapenem in Combination With Xuebijing Injection Against Sepsis
Source: Front Pharmacol. 2020 Apr 22;11:502. doi: 10.3389/fphar.2020.00502 (PMC7189733; doi:10.3389/fphar.2020.00502)
Supplement: Supplementary file 5 [file Table_4.docx]

**Supplementary Table S4** The concentrations of 14 dominating compounds in xuebijing injection and corresponding precision, repeatability, stability and recovery.

| **Components** | **Concentration  Mean ± SD (μg/mL)** | **Precision  RSD (%)** | **Repeatability**  **RSD (%)** | **Stability  RSD (%)** | **Recovery Mean ± SD (%)** |
| --- | --- | --- | --- | --- | --- |
| Hydroxysafflor yellow A | 594.19 ± 7.18 | 2.13 | 4.15 | 3.41 | 100.40 ± 1.60 |
| Oxypaeoniflorin | 38.48 ± 1.95 | 2.80 | 3.35 | 3.86 | 98.86 ± 3.12 |
| Benzoylpaeoniflorin | 29.68 ± 0.45 | 2.11 | 3.79 | 2.39 | 101.44 ± 3.04 |
| Senkyunolide I | 21.38 ± 0.60 | 1.22 | 4.04 | 3.20 | 98.99 ± 2.63 |
| Succinic acid | 14.54 ± 0.64 | 2.35 | 2.57 | 2.91 | 100.24 ± 2.75 |
| Gallic acid | 6.53 ± 0.18 | 2.57 | 1.41 | 2.87 | 100.22 ± 2.79 |
| Rosmarinic acid | 5.32 ± 0.16 | 1.91 | 3.70 | 2.01 | 100.23 ± 2.48 |
| Caffeic acid | 4.83 ± 0.22 | 1.22 | 3.74 | 2.06 | 99.11 ± 2.48 |
| Protocatechuic aldehyde | 4.41 ± 0.10 | 3.30 | 4.89 | 2.98 | 100.74 ± 3.27 |
| Protocatechuic acid | 4.19 ± 0.11 | 3.82 | 3.94 | 3.17 | 98.25 ± 1.96 |
| Rutin | 3.43 ± 0.15 | 2.96 | 3.64 | 2.58 | 101.49 ± 2.88 |
| Salvianic acid A | 3.09 ± 0.14 | 1.63 | 2.59 | 3.88 | 99.03 ± 2.48 |
| Chlorogenic acid | 2.98 ± 0.07 | 2.64 | 3.66 | 3.27 | 99.39 ± 1.95 |
| Naringenin | 0.34 ± 0.01 | 2.44 | 4.60 | 2.61 | 100.31 ± 2.52 |
